# Comparison of Resampling Techniques for Imbalanced Datasets in Machine Learning: Application to Epileptogenic Zone Localization From Interictal Intracranial EEG Recordings in Patients With Focal Epilepsy

**DOI:** 10.3389/fninf.2021.715421

**Published:** 2021-11-19

**Authors:** Giulia Varotto, Gianluca Susi, Laura Tassi, Francesca Gozzo, Silvana Franceschetti, Ferruccio Panzica

**Affiliations:** ^1^Epilepsy Unit, Bioengineering Group, Fondazione IRCCS Istituto Neurologico Carlo Besta, Milan, Italy; ^2^Neurophysiopathology Unit, Fondazione IRCCS Istituto Neurologico Carlo Besta, Milan, Italy; ^3^Universidad Complutense de Madrid-Universidad Politécnica de Madrid (UPM-UCM) Laboratory of Cognitive and Computational Neuroscience, Center of Biomedical Technology, Technical University of Madrid, Madrid, Spain; ^4^Department of Experimental Psychology, Cognitive Processes and Logopedy, Complutense University of Madrid, Madrid, Spain; ^5^“Claudio Munari” Epilepsy Surgery Centre, Niguarda Hospital, Milan, Italy; ^6^Clinical Engineering, Fondazione IRCCS Istituto Neurologico Carlo Besta, Milan, Italy

**Keywords:** imbalanced dataset classification, re-sampling techniques, oversampling and undersampling, ensemble methods, network analysis, epilepsy surgery, stereo-EEG/intracranial recordings, epileptogenic zone localization

## Abstract

**Aim:** In neuroscience research, data are quite often characterized by an imbalanced distribution between the majority and minority classes, an issue that can limit or even worsen the prediction performance of machine learning methods. Different resampling procedures have been developed to face this problem and a lot of work has been done in comparing their effectiveness in different scenarios. Notably, the robustness of such techniques has been tested among a wide variety of different datasets, without considering the performance of each specific dataset. In this study, we compare the performances of different resampling procedures for the imbalanced domain in stereo-electroencephalography (SEEG) recordings of the patients with focal epilepsies who underwent surgery.

**Methods:** We considered data obtained by network analysis of interictal SEEG recorded from 10 patients with drug-resistant focal epilepsies, for a supervised classification problem aimed at distinguishing between the epileptogenic and non-epileptogenic brain regions in interictal conditions. We investigated the effectiveness of five oversampling and five undersampling procedures, using 10 different machine learning classifiers. Moreover, six specific ensemble methods for the imbalanced domain were also tested. To compare the performances, Area under the ROC curve (AUC), F-measure, Geometric Mean, and Balanced Accuracy were considered.

**Results:** Both the resampling procedures showed improved performances with respect to the original dataset. The oversampling procedure was found to be more sensitive to the type of classification method employed, with Adaptive Synthetic Sampling (ADASYN) exhibiting the best performances. All the undersampling approaches were more robust than the oversampling among the different classifiers, with Random Undersampling (RUS) exhibiting the best performance despite being the simplest and most basic classification method.

**Conclusions:** The application of machine learning techniques that take into consideration the balance of features by resampling is beneficial and leads to more accurate localization of the epileptogenic zone from interictal periods. In addition, our results highlight the importance of the type of classification method that must be used together with the resampling to maximize the benefit to the outcome.

## Introduction

Epilepsy is a chronic neurological disease affecting 1% of the worldwide population (Fiest et al., 2017). Approximately 30% of the patients with focal epilepsies are resistant to the antiepileptic drugs (AEDs), and they can be considered as candidate for epilepsy surgery, with the aim of removing the epileptogenic zone (EZ). The latter is defined as the minimum amount of cortex that must be resected (inactivated or completely disconnected) to produce seizure freedom (Lüders et al., 2006; Ryvlin et al., 2014). However, the correct localization of the EZ to achieve seizure freedom after surgery, is still an unsolved and open question, as indicated by the high rate of failure of seizure control (30–40%) after surgery (Spencer and Huh, 2008; Bulacio et al., 2012). The advanced signal processing approaches, especially those based on the connectivity analysis, have been largely applied to stereo-electroencephalography (SEEG) from the patients with epilepsy to better pinpoint the location of the EZ (Varotto et al., 2013; Bartolomei et al., 2017; Adkinson et al., 2019; Narasimhan et al., 2020).

The supervised machine learning methods are increasingly applied in epilepsy research, representing useful tools to integrate the complex and large-scale data deriving from different electrophysiological or imaging techniques, such as EEG, magnetoencephalography (MEG), functional-MRI (fMRI), or positron emission tomography (PET) (refer to Abbasi and Goldenholz, 2019 for a comprehensive review). Most of these studies focused on the following aspects: diagnosis of epilepsy (Kassahun et al., 2014; Azami et al., 2016; Soriano et al., 2017), seizure prediction (Acharya et al., 2018; Kiral-Kornek et al., 2018; Daoud and Bayoumi, 2019), lateralization of temporal lobe epilepsy (Jin and Chung, 2017; Frank et al., 2018; Peter et al., 2018), and post-surgical outcome prediction (Armañanzas et al., 2013; Goldenholz et al., 2016; Gleichgerrcht et al., 2018). With respect to the localization of the EZ and support to pre-surgical planning, few works applied machine learning tools, showing the promising usefulness of this approach, and the need for further investigation and generalization (Dian et al., 2015; Elahian et al., 2017; Khambhati et al., 2017; Roland et al., 2017). In this specific framework, one central issue that should be taken into account, and which could represent one of the main limitations, is that the EZ represents a smaller region compared with the other non-EZ areas explored. This leads to an uneven distribution of the majority (non-EZ) and minority (EZ) classes, which can strongly worsen or limit the classification performances. This situation is known as the class imbalance problem and can be considered one of the central topics in machine learning research (He and Garcia, 2009; Ali et al., 2015; Fernández et al., 2018).

In the past decade, many different approaches have been developed to cope with imbalanced classification, most of them based on four different families: resampling techniques, cost-sensitive learning, algorithm modification, and ensemble methods (Mena and Gonzalez, 2006; Galar et al., 2012; Krawczyk et al., 2014; Loyola-González et al., 2016).

Among these, the methods belonging to the data resampling family have been proved useful as well as relatively simple approaches to be applied in the medical context (Lee, 2014; Loyola-González et al., 2016). In data resampling, the training instances are modified to rebalance the class distribution through *oversampling* of the minority class, or *undersampling* of the majority one, before training the classifier. Oversampling could have the limitation of overfitting the minority class, while undersampling could eliminate potential useful information for correct classification (Chawla, 2009).

Different studies dealt with the comparisons of performances of most of the existing resampling techniques, most of which were applied to a wide variety of datasets together, being mainly aimed at assessing the robustness of results across different dataset combinations (López et al., 2013). Nevertheless, when applied to a single specific dataset, such comparison can lead to different results (Xie et al., 2020), reflecting a lack of consensus about the performances of such techniques and putting in evidence the need for *ad-hoc* comparisons in each specific clinical framework.

To the best of our knowledge, this is the first study focused on the evaluation and comparison of these approaches in the context of epilepsy, and in particular, in the framework of the surgical planning based on analysis of electrophysiological intracranial recordings.

In this study, we compared five oversampling and five undersampling procedures and tested the resulting rebalanced datasets with 10 different machine learning classifiers (such as both standard machines and classical ensemble approaches). Moreover, six specific ensemble methods properly modified for imbalanced domain and belonging to data variation-based ensemble were tested and compared. In these algorithms, the resampling phase is applied to each step of the ensemble classifier, in such a way that each classifier is trained with a different resampled dataset (Galar et al., 2012). For this reason, we considered them as an extension of resampling methods, which need to be compared with the oversampling and undersampling techniques combined with the classical ensemble approaches.

The classification was based on the features obtained by network analysis of interictal SEEG recorded from the 10 patients who underwent epilepsy surgery and were seizure-free (SF) after 3 years of follow-up.

To compare the performances, *area under the ROC curve* (AUC), *balanced accuracy* (BalACC), *F-measure* (Fm), and *geometric mean* (Gmean) were used as metrics, since these are usually considered suitable measures to deal with the imbalanced datasets (Bekkar et al., 2013; López et al., 2013).

## Materials and Methods

**Figure 1:**
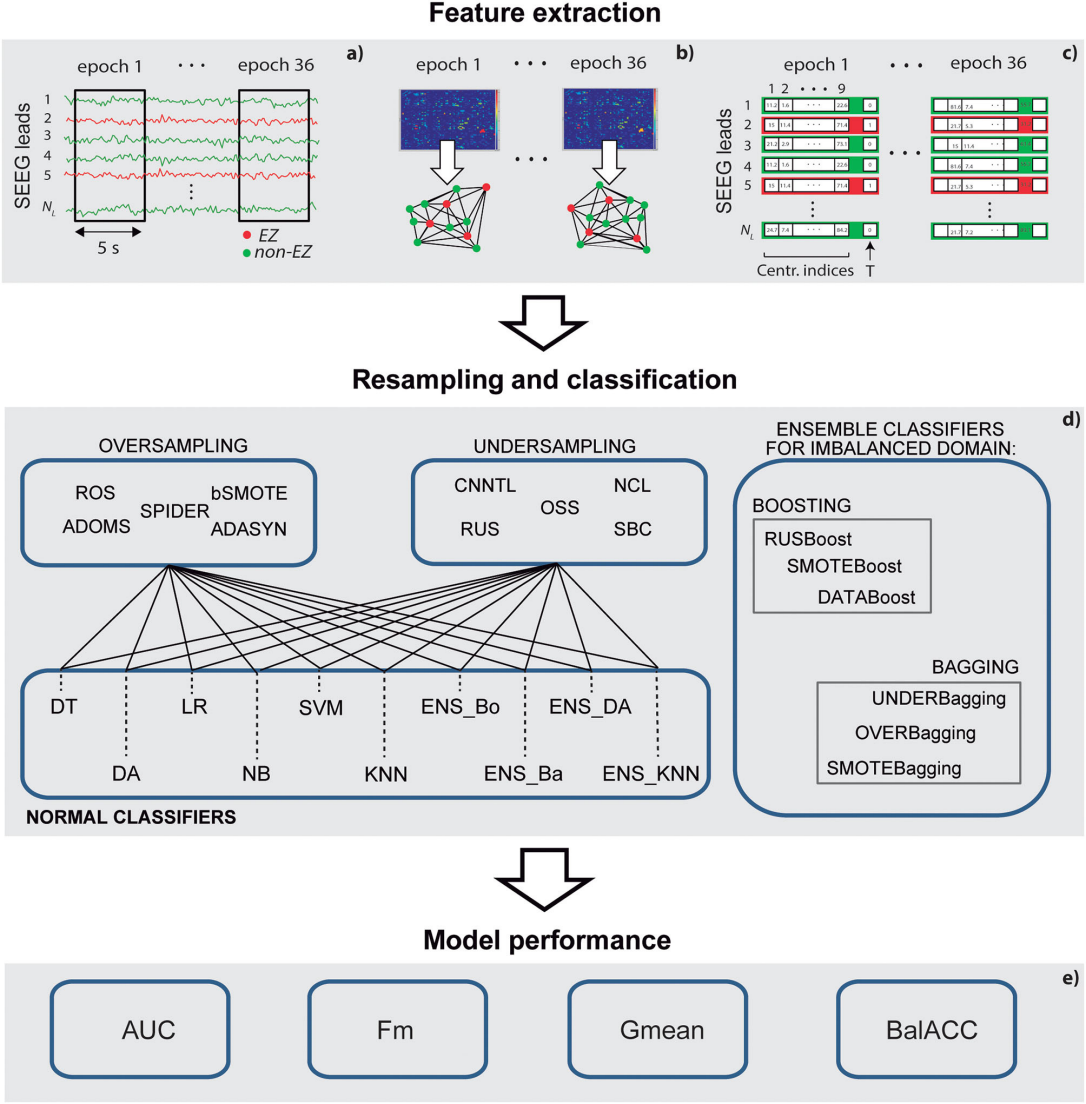
A schematic representation of the methodological pipeline. **(a)** Stereo-electroencephalography (SEEG) epochs selection; **(b)** connectivity analysis and graph-based indexes of centrality calculation; **(c)** feature selection and training and test set definition; **(d)** set of resampling methods and classifiers applied; and **(e)** measures to assess the performance of the models.

We start this section by describing the steps of selection and signal recording of the patients. The methodological pipeline is then outlined: feature extraction, data resampling, classification, and evaluation of the performance of the model (as shown in Figure 1 for a schematic representation). Finally, we describe the statistical analysis, which has been performed to evaluate the consistency of our results.

### Selection of Patients

The study involved SEEG signals recorded from *Np* = 10 patients (three women) with drug-resistant focal epilepsy at the Claudio Munari Epilepsy Surgery Center of Niguarda Hospital (Milan, Italy). The patients were selected from the 41 patients implanted with SEEG electrodes over 24 months. Among them, 24 had negative MRI and 10 of them were seizure-free after at least 3 years of follow-up and were finally considered for this study. Table 1 presents the details of the main clinical features.

**Table 1:** Main clinical features, epileptogenic zone (EZ) localization performed by the standard methods, and surgery outcomes for the patients enrolled in this study.

**Id**	**Gender**	**Age**	**Onset**	**Sz/m**	**Side**	**Lobe**	**Histology**	**Follow-Up/M**	**AEDs**
1	M	27	19	10	R	TFI	crypto	68	Reduced
2	M	25	4	10	L	TI	crypto	48	Stopped
3	F	30	16	5	R	T	crypto	54	Reduced
4	F	27	17	10	R	T	crypto	46	Stopped
5	M	40	16	30	L	F	no	70	Reduced
6	M	39	20	1	R	F	crypto	42	Reduced
7	M	28	11	3	L	F	crypto	65	Ongoing
8	M	22	16	15	L	TO	crypto	34	Reduced
9	M	44	22	10	R	TO	FCD Ib	62	Reduced
10	F	35	4	5	R	TPCF	FCD Ia	71	Ongoing

*AEDs, antepileptic drugs; crypto, cryptogenic; FCD, focal cortical dysplasia; F, female; FC, fronto-central; FCD, focal cortical dysplasia; Fr, Frontal; HS, hippocampal sclerosis; M, male; Sz//m, seizures per month; Age, age at surgical intervention; Onset, age of epilepsy onset; PCI, parieto-centro-insular; T, Temporal; TFI, temporo-fronto-insular; TI, temporo insular; TC, temporo-central; TO, temporo-occipital; TPCF, temporo-parieto-centro-frontal. AEDs column refers to variation of drug therapy with respect to pre-surgical condition*.

The mean age of the patients was 31.7 ± 7.3 years, and the mean duration of epilepsy was 17.2 ± 7.8 years. They had no obvious risk factor for epilepsy. The surgical outcome was assessed after at least 3 years of follow-up after surgery (mean follow-up period: 56 ± 13 months) and classified as class I according to Engel's classification (Engel, 1993).

### SEEG Recordings

Stereo-electroencephalography signals were recorded using the multi-lead platinum-iridium electrodes (Dixi, Besançon, France, with 5–18 contacts of diameter 0.8 mm; 1.5 mm long; and 2 mm apart), implanted under general anesthesia after stereo-arteriography using a 3D MRI imported into a computer-assisted neuronavigational module to localize the blood vessels and guide electrode trajectory. The placement of intracerebral electrodes was defined according to the data derived by non-invasive anatomo-electroclinical procedures (Talairach and Bancaud, 1966; Cardinale et al., 2019).

The SEEG signals were recorded using a common reference electrode (Nikon-Kohden system; 192-channels; sampling rate 1 kHz) under video and clinical control over 5–20 days and then examined by the two expert neurologists to define the EZ and plan the surgical approach and resection. EZ was defined by considering ictal discharge recordings, responses associated with the intracerebral electrical stimulations, and neurophysiological mapping, and then integrated into the definition of the brain area(s) to be surgically excised. Post-resection MRI was used to identify the areas of the brain that were effectively removed. The target value to assess the classification performances—SEEG leads as belonging to EZ or non-EZ—was defined by considering the intersection between the group of SEEG leads labeled as EZ by the clinicians through the pre-surgical evaluation, and the resected zone.

### Feature Extraction

Stereo-electroencephalography signals were analyzed using bipolar derivations, and those presenting non-physiological artifacts were excluded from the analysis. The number of analyzed SEEG leads differed for each patient being on average *N*_*L*_ = 73 ± 6. Furthermore, 3 min of continuous interictal SEEG signals, recorded during awake condition at least 1 h far from any ictal event, were selected and divided into *N*_*E*_ = 36, five s length, non-overlapping epochs. After testing several lengths and epochs partitions, 3 min length was selected as the minimum recording time to obtain a good EZ classification. The broad 1–80 Hz frequency band was used for the analysis. In addition, 36 time-varying connectivity matrices were estimated by applying a bivariate non-linear method and the non-linear regression index (h2) (Lopes da Silva et al., 1989; Wendling et al., 2010) (refer to Supplementary Material). In this regard, a wide variety of methods have been proposed to estimate the SEEG connectivity, all of them being characterized by different advantages and pitfalls strongly depending on the signal and the aim of the study (Silfverhuth et al., 2012; Olejarczyk et al., 2017). Among them, a non-linear regression analysis has been proved to be particularly suitable to estimate the connectivity from the simulated coupled neuronal population (Wendling et al., 2009), and has been largely applied in the specific contest of intracranial EEG recordings and EZ localization (Bartolomei et al., 2017).

From the adjacency matrices, the corresponding graphs were built for each patient, after applying a threshold to select the minimum number of connections that ensures a connected graph for all the epochs.

After a preliminary analysis involving several graph theory-based indices, nine of them, focusing on different complementary network properties of centrality (Oldham et al., 2019), were identified as the optimal one to classify EZ in the whole group of patients, and used as features of the classifier: *outdegree centrality (Ce), indegree Ce, oustrength Ce, instrength Ce., betweenness Ce., outcloseness Ce., incloseness Ce., pagerank Ce.*, and *eigenvector Ce*. (as shown in Supplementary Material for a detailed description of the basic properties of these metrics).

The connectivity analysis was performed through a specific custom-written toolbox developed in Matlab (R20a; MathWorks Inc., Natick, MA, USA). Matlab graph toolbox and the Brain connectivity toolbox (Rubinov and Sporns, 2010), were used for graph analysis.

To provide the classifiers with a suitable number of trials, we first grouped all the values of the features pertaining to the different time epochs and obtained, for each patient *p*, a matrix with *N*_*L,p*_ × *N*_*E*_ rows and 10 columns (i.e., nine features and one target). EZ has been considered as the positive class, with 1 indicating the EZ class and 0 the non-EZ class. The imbalanced ratio (IR)—the ratio between the number of trials pertaining to positive and negative classes—for each patient, is indicated in Table 2.

**Table 2:** Number of analyzed SEEG leads (Total SEEG leads), number of leads belonging to the EZ (EZ leads), and Imbalanced ratio (IR) per patient.

**Pt id**	**Tot SEEG leads**	**EZ leads**	**IR**
1	66	5	12.2
2	73	6	11.2
3	80	7	10.4
4	81	9	8.0
5	62	4	14.5
6	72	2	35.0
7	76	8	8.5
8	78	13	5.0
9	72	5	13.4
10	72	7	9.3

Since one of the main objectives of the proposed procedure was to classify SEEG signals of every single patient independently from the others, training and test set were defined by considering a proportion of 9:1, using features from nine patients for training and features from one single patient for test. For further statistical analysis, the same splitting was repeated for all the combinations of patients, thus providing 10 different training-testing datasets.

### Data Resampling

In all the patients, more electrode contacts were implanted in the non-epileptogenic than epileptogenic regions. This fact is reflected in a smaller number of EZ trials than the non-EZ trials, giving rise to the problems with the statistics of the applied classification methods (and hence, the subsequent learning by machine learning models).

Among the existing resampling techniques to tackle such class imbalance problems, we selected five methods of oversampling and five methods of undersampling and compared the performance of classifiers with respect to the original dataset.

The oversampling methods are based on the creation of a new bigger dataset, obtained by replicating or creating new samples, usually from the minority class:

- *Adaptive Synthetic Sampling* (ADASYN). ADASYN generates data considering a weighted distribution for different minority class examples, where more synthetic data are generated for minority class examples that are harder to learn compared with those easier to learn (He et al., 2008).
- *Adjusting the direction of the synthetic minority class example* (ADOMS). ADOMS generates positive data instances from other instances in the original dataset selecting k as the nearest neighbors and using them to perform arithmetical operations to generate the new instance by principal component analysis (PCA) (Tang and Chen, 2008).
- *Random oversampling* (ROS). ROS generates minority class instances randomly (Batista et al., 2004).
- Selective Pre-processing for Imbalanced Data (SPIDER). SPIDER oversamples instances from the minority class that are difficult to learn and, at the same time, filters the examples from the majority class which are also difficult to learn (Stefanowski and Wilk, 2008).
- *Borderline-Synthetic Monitoring Oversampling Technique* (bSMOTE). The bSMOTE generates positive data instances from other instances in the original dataset selecting k as the nearest neighbors and using them to perform the arithmetical operations to generate the new instance (Han et al., 2005).

The undersampling methods are based on the reduction of the original dataset by eliminating samples, usually form the majority class:

- Condensed Nearest Neighbor + Tomek's modification of Condensed Nearest Neighbor (CNNTL). CNNTL applies the CNN method and the Tomek Links method in a chain to delete the instances that lead us to misclassify new instances in the imbalanced domains (Batista et al., 2004).
- *Neighborhood Cleaning Rule* (NCL). NCL finds a subset S of the training set T applying the neighborhood cleaning rule of examples (Laurikkala, 2001).
- *One Side Selection* (OSS). OSS finds a subset S of the training set T applying the OSS of examples (Kubat and Matwin, 1997).
- *Random Undersampling* (RUS). RUS deletes the majority of class data instances randomly (Batista et al., 2004).
- *Undersampling based on clustering* (SBC). After dividing all the training samples into some clusters, SBC selects a suitable number of majority class samples from each cluster by considering the ratio of the number of majority class samples to the number of minority class samples in the cluster (Yen and Lee, 2006).

For both oversampling and undersampling methods, the default parameters were used. The corresponding parameters set can be found in the method library of KEEL software (UGR Granada, Spain) (Alcalá-Fdez et al., 2011).

### Classification

To classify and compare the different resampled datasets, 10 different machine learning algorithms, belonging to the family of supervised classification, and most used in the contest of neurophysiological signal processing, were applied as follows:

1. Decision tree (DT): coarse tree, whose maximum number of branch points is set to 4. The method adopts the Gini's diversity index as the split criterion and envisages a pruning procedure.
2. Discriminant analysis (DA): creates non-linear boundaries between the classes (quadratic discriminant analysis).
3. Logistic regression (LR).
4. Naïve Bayes (NB): the method supports continuous attributes by assuming a Gaussian distribution (Gaussian Naïve Bayes).
5. Support vector machine (SVM): characterized by coarse distinctions between the classes, with kernel scale set to $4\sqrt{P}$, where *P* is the number of predictors (Coarse Gaussian SVM).
6. KNN (K-nearest neighbors): where we set the number of neighbors to 100 (Coarse distinctions between classes) and used the Euclidean distance metric (coarse KNN).
7. Boosted Ensemble (EnsBO): ensemble classifier which uses the meta-algorithm AdaBoost (Freund and Schapire, 1999).
8. Bagged Ensemble (EnsBA), Random forest Bag, with DT learners. This implementation uses Breiman's “random forest” algorithm (Breiman, 2001).
9. Discriminant Analysis Ensemble (EnsDA): combines different feature subsets to improve the classification performance (subspace ensemble), and uses Discriminant learners.
10. KNN ensemble (EnsKNN): Subspace ensemble with Nearest Neighbor learners.

During the training phase, the validation step was performed through a 5-fold cross-validation approach. For all the considered methods, default parameters were used. The corresponding parameters set can be found in the *Matlab classification learner* toolbox specification.

### Ensemble Methods for Imbalanced Domain

Since the main objective of the study was to compare the effect of different resampling techniques on the classifier performances, in the previous section we described both the standard and classical ensemble classifiers, with the resampling procedure applied before the classification.

However, in the past years, ensemble-based classifiers have been considered a suitable approach in the imbalanced domain, leading to the implementation of specific modification of the ensemble algorithm, in which the data rebalancing pre-processing is integrated into the ensemble algorithm and done before the learning stage of each classifier of the ensemble (Chawla et al., 2003; Seiffert et al., 2010). For this reason, we also tested six of these approaches, three belonging to boosting (methods 1–2–3) and three to bagging (methods 4–5–6) approach:

1. DATABoost: it combines the AdaBoost algorithm with a data generation strategy. It first identifies hard examples (seeds) and then carries out a rebalance process, always for both the classes (Guo and Viktor, 2004).
2. RUSBoost: multi-class AdaBoost with RUS in each iteration (Seiffert et al., 2010).
3. SMOTEBoost: multiclass AdaBoost with SMOTE in each operation (Chawla et al., 2003).
4. OVERBag: bagging with oversampling of the minority class (Wang and Yao, 2009).
5. SMOTEBag: bagging where SMOTE quantity of each bag varies (Wang and Yao, 2009).
6. UnderBag: bagging with undersampling of the majority class (Barandela et al., 2003b).

### Performances Metrics

In common practice, accuracy is the most used measure to assess classifier performance. However, since it does not allow to distinguish between the number of correctly classified instances of the two different classes, it can lead to an erroneous conclusion when applied in the context of imbalanced datasets.

To assess and compare the performances of the classifiers, we used the following four metrics, which have been proven to be suitable for the imbalanced domain (Bekkar et al., 2013; López et al., 2013; Fernández et al., 2018):

$$\begin{array}{l} AUC=\frac{1+TPr+FPr}{2}\\ Fm=\frac{\left(1+\beta^{2}\right)\left(PPV\cdot TPr\right)}{\beta^{2}\cdot PPV+TPr)}\\ GMean=\sqrt{\frac{TP}{TP+FN}\cdot \frac{TN}{FP+TN}}\\ BalACC=\frac{TPr+TNr}{2} \end{array}$$

Where TPr is the *true positive rate* (or *sensitivity)*, TNr is the *true negative rate* (or *specificity)*, and PPV is the *positive predicted value*, respectively, defined as:

$$\begin{array}{l} TPr=\frac{TP}{TP+FN}\\ TNr=\frac{TN}{TN+FP}\\ PPV=\frac{TP}{TP+FP} \end{array}$$

Note that TP, TN, FP, and FN stay for true positives, true negatives, false positives, and false negatives, respectively. For Fm we used β = 1, to assign equal importance to both TP and PPV.

All the analyses were performed using the *KEEL* software (Alcalá-Fdez et al., 2011) and the *Matlab classification learner* toolbox.

### Statistical Analysis

To compare the different resampling techniques, Friedman's test was applied to the four performances metrics AUC, Fm, Gmean, and BalACC (Friedman, 1937). When a significant difference among the group was found, Shaffer's *post-hoc* test was applied for multiple comparisons (Shaffer, 1986). The alpha level for statistical significance was set at 0.05, and the final adjusted *p*-values are used for the results. All the statistical comparisons were performed using SPSS (IBM Corp. Version 26.0. Armonk, NY, USA) and KEEL software.

Data are available from the corresponding authors upon request.

## Results

### Oversampling

**Figure 2:**
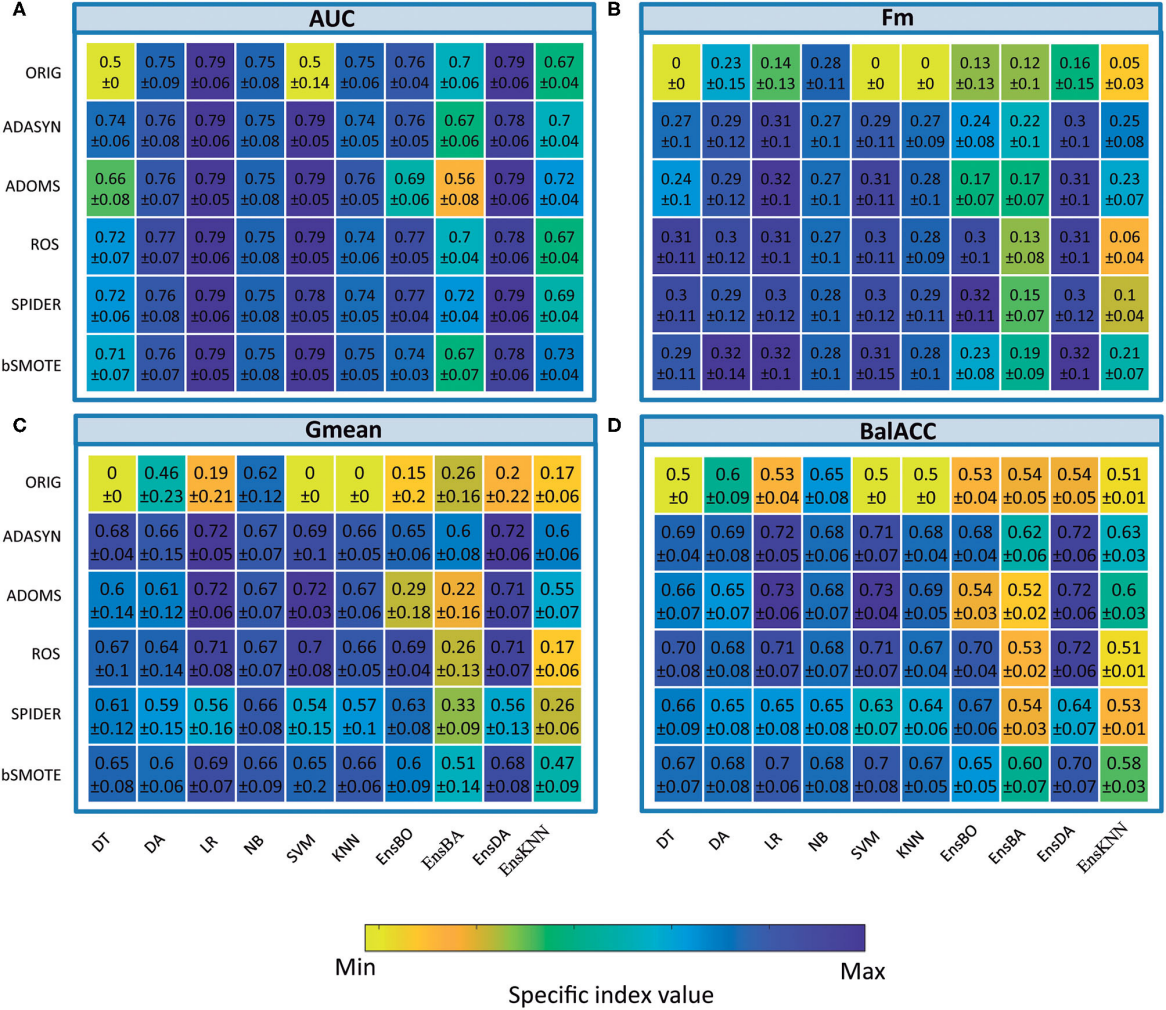
Comparison of classifier performances (mean and SD values), among the different oversampling procedures, in terms of indices Area under the ROC curve (AUC) **(A)**, F-measure (Fm) **(B)** Geometric Mean (Gmean) **(C)**, and Balanced Accuracy (BalACC) **(D)**. *X*-axis: classifiers applied; *Y*-axis: resampling techniques. For ease of understanding, the colormap spans from minimum to maximum values of each specific index. As shown in Table 2 and Supplementary Tables 2–5 for statistical comparisons among these values.

The average predicted performances in terms of AUC, Fm, Gmean, and BalACC are shown in Figure 2. For all 10 classifiers, the statistical results of the Friedman's Test and related Shaffer's *post-hoc* comparisons for AUC (a), Fm (b), Gmean (c), and BalACC (d) are shown in Table 3. Shaffer's *post-hoc* comparisons have been indicated only when Friedman's test resulted significantly. The sign “–” (respectively, “+”) indicates that the first algorithm has a lower (higher) value than the second one.

- *The area under the ROC curve*: Friedman's test revealed significant differences among the pre-processing techniques only in five of the classifiers tested (DT, SVM, Ens_BO, Ens_BA, and Ens_KNN). For the two standard classifiers (DT and SVM), the *post-hoc* comparisons revealed differences only with respect to the original datasets, while no differences were present among the five oversampling techniques. Interestingly, for three of the four classical ensemble classifiers, none of the resampling techniques performed better than the original dataset. On the contrary, the ADOMS approach showed significantly lower AUC values than the other methods in both boosted and bagged ensemble classifiers. In the KNN ensemble, both original and ROS datasets reported the lowest performances (as shown in Table 3 and Supplementary Table 2).
- *F-measure*: the significant differences have been revealed in 8 out of the 10 classifiers (DT, LR, SVM, KNN, EnsBO, EnsBA, EnsDA, and EnsKNN). The *post-hoc* comparisons showed the lower performance of the original dataset with respect to all resampling procedures in the six standard classifiers. In the ensemble both original and ADOMS had significantly lower Fm values than the other algorithm ms (as shown in Table 3 and Supplementary Table 3).
- *Geometric Mean*: this metric exhibited more differences among the considered resampling approaches. All the classifiers except LR showed significant differences among the rebalancing approaches. In the standard classifiers and the EnsDA, the algorithm ADASYN, ADOMS, ROS, and bSMOTE performed better than both the original and SPIDER dataset. As for Fm, in Boosted and Bagged and KNN Ensemble ADOMS algorithm reported the lowest performance (as shown in Table 3 and Supplementary Table 4).
- *Balanced Accuracy*: significant differences among the different resampling algorithms emerged for all the 10 classifiers. According to Shaffer's *post-hoc* analysis, ADASYN, ADOMS, ROS, and bSMOTE reported better performances than the original and SPIDER datasets in the standard classifiers. In the EnsBO and EnsBA, no differences were found between the original and ADOMS data set, which performed worse than the other resampling procedures. In the EnsDA classifier, the resampling algorithms ADASYN, ADOMS, ROS, and bSMOTE showed higher BalACC than the original and SPIDER dataset. In EnsKNN classifier, showed similar results than EnsDA, except for ROS, which reported BalACC comparable with original and SPIDER (as shown in Table 3 and Supplementary Table 5).

**Table 3:** Friedman's and *post-hoc* Shaffer's test for the *oversampling* techniques applied to the four performance measures: Area under the ROC Curve (AUC), F-measure (Fm), Geometric Mean (Gmean), and Balanced Accuracy (BalACC).

**Oversampling**		***DT***	***DA***	***LR***	***NB***	***SVM***	***KNN***	***EnsBO***	***EnsBA***	***EnsDA***	***EnsKNN***
Original vs. *ADASYN*	AUC	**–**				**–**		**–**			**–**
	Fm	**–**						**–**	**–**		**–**
	Gmean	**–**	**–**	**–**		**–**	**–**	**–**	**–**	**–**	**–**
	BalACC	**–**	**–**	**–**	**–**	**–**	**–**	**–**	**–**	**–**	**–**
Original vs. *ADOMS*	AUC					**–**			**+**		**–**
	Fm			**–**		**–**	**–**				
	Gmean	**–**		**–**		**–**	**–**			**–**	**–**
	BalACC	**–**		**–**	**–**	**–**	**–**			**–**	**–**
Original vs. *ROS*	AUC	**–**				**–**					
	Fm	**–**		**–**		**–**	**–**	**–**		**–**	**–**
	Gmean	**–**	**–**	**–**		**–**	**–**	**–**		**–**	
	BalACC	**–**	**–**	**–**	**–**	**–**	**–**	**–**		**–**	
Original vs. *SPIDER*	AUC	**–**									
	Fm	**–**		**–**		**–**	**–**	**–**		**–**	**–**
	Gmean							**–**			
	BalACC				**–**			**–**			
Original vs. *bSMOTE*	AUC					**–**					**–**
	Fm	**–**		**–**		**–**	**–**			**–**	**–**
	Gmean	**–**	**–**	**–**		**–**	**–**	**–**	**–**	**–**	**–**
	BalACC	**–**	**–**	**–**		**–**	**–**	**–**		**–**	**–**
*ADASYN* vs. *ADOMS*	AUC							**+**			
	Fm										
	Gmean							**+**	**+**		
	BalACC							**+**	**+**		
*ADASYN* vs. *ROS*	AUC										
	Fm								**+**		
	Gmean								**+**		**+**
	BalACC								**+**		**+**
*ADASYN* vs. *SPIDER*	AUC										
	Fm										
	Gmean			**+**						**+**	**+**
	BalACC			**+**						**+**	**+**
*ADASYN* vs. *bSMOTE*	AUC										
	Fm										
	Gmean										
	BalACC										
*ADOMS* vs. *ROS*	AUC							**–**	**–**		**+**
	Fm							**–**			
	Gmean							**–**			**+**
	BalACC							**–**			**+**
*ADOMS* vs. *SPIDER*	AUC							**–**	**–**		
	Fm							**–**			
	Gmean			**+**			**+**			**+**	
	BalACC			**+**			**+**	**–**		**+**	
*ADOMS* vs. *bSMOTE*	AUC										
	Fm										
	Gmean								**–**		
	BalACC								**–**		
*ROS* vs. *SPIDER*	AUC										
	Fm										
	Gmean			**+**						**+**	
	BalACC			**+**						**+**	
*ROS* vs. *bSMOTE*	AUC										**–**
	Fm										
	Gmean								**–**		**–**
	BalACC										**–**
*SPIDER* vs. *bSMOTE*	AUC										
	Fm										
	Gmean										
	BalACC										

*The 10 columns refer to the 10 classifiers models. The comparisons showing significant results are indicated with a “–” sign when the first algorithm (of the two compared in each row) was lower or with a “+” sign when it was higher than the second one. The rows without significant differences are not reported. Complete results with the p-values can be found in Supplementary Tables 3–6*.

### Undersampling

**Figure 3:**
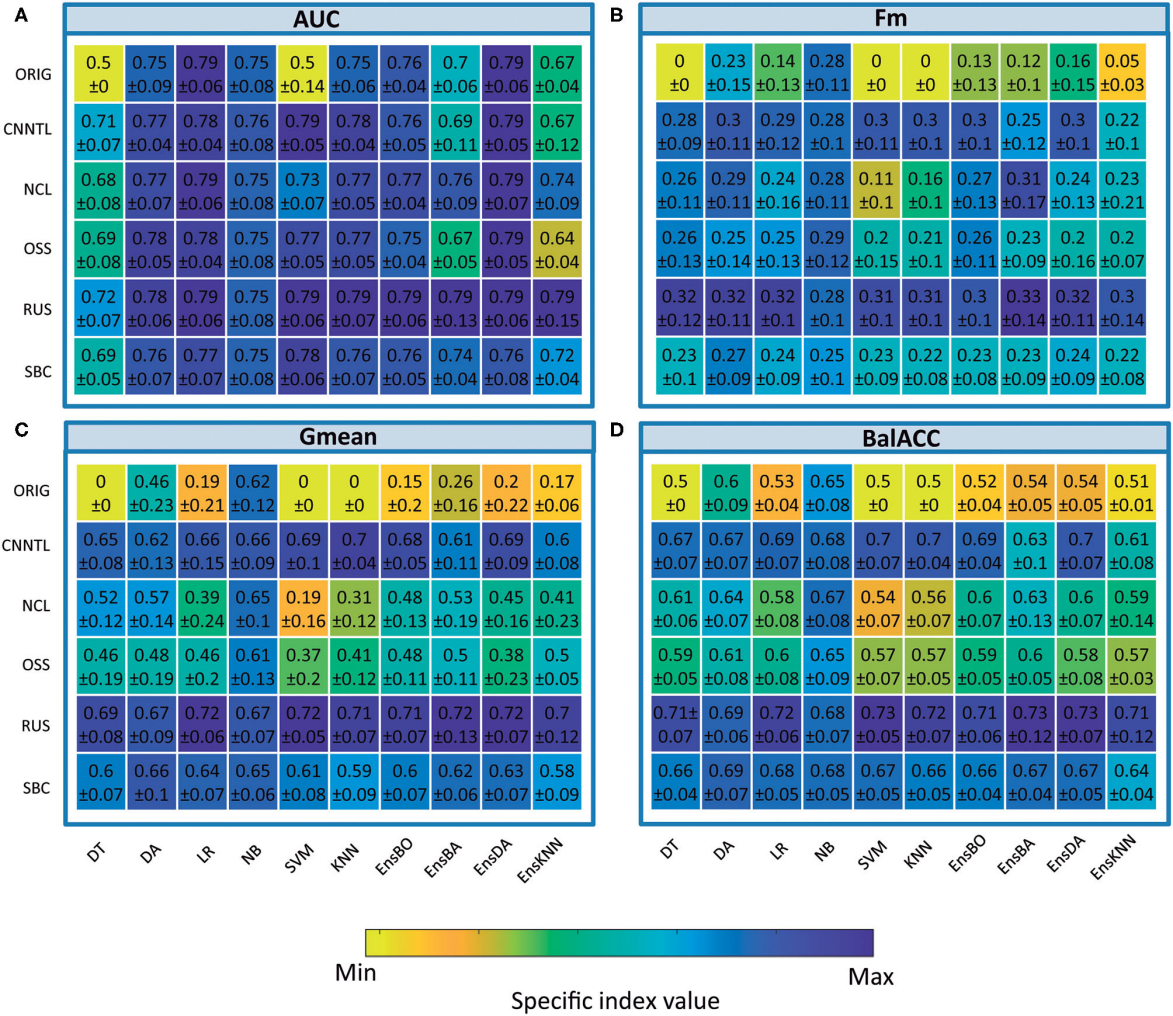
Comparison of classifier performances (mean and SD), among the different undersampling procedures, in terms of indices AUC **(A)**, Fm **(B)**, Gmean **(C)**, and BalACC **(D)**. *X*-axis: classifiers applied; *Y*-axis: resampling techniques. For ease of understanding, the colormap spans from minimum to maximum values of each specific index. As shown in Table 3 and Supplementary Tables 6–9 for statistical comparisons among these values.

The average predicted performances of undersampling procedures in terms of AUC, Fm, Gmean, and BalACC are shown in Figure 3. For all the 10 classifiers, the statistical results of the Friedman's Test and related Shaffer's *post-hoc* comparisons for AUC (a), Fm (b), Gmean, (c), and BalACC (d) are shown in Table 4, respectively. Shaffer's *post-hoc* comparisons have been indicated only when Friedman's Test resulted significantly; The sign “–” (respectively “+”) indicates that the first algorithm has a lower (higher) value than the second one.

- The area under the ROC curve: significant differences among the pre-processing techniques are found in five of the classifiers tested (DT, SVM, KNN, EnsBA, and EnsKNN). In the DT classifier, all undersampling algorithms performed equally and better than the original one; in SVM, RUS, and CNNTL performed better than the others, and in KNN only RUS showed improved AUC performances with respect to the original and all the other resampling techniques. In EnsBA and EnsKNN, significantly improved performances were achieved by NCL, RUS, and SBC (as shown in Table 4 and Supplementary Table 6).
- *F-measure*: Friedman's test revealed significant differences in 9 out of the 10 classifiers (all except NB). For standard classifiers, *post-hoc* comparisons showed the lower performance of the original dataset with respect to all resampling procedures except for SBC in DT classifier, NCL in SVM and KNN, and NCL, OSS, and SBC in LR classifier. As well as in standard classifiers, also in all the ensembles, the best performances were achieved by RUS, followed by the CNNTL algorithm (as shown in Table 4 and Supplementary Table 7).
- *Geometric Mean* showed significant differences among the considered approaches for all the classifiers, proving to be more suited than AUC and Fm in capturing the differences among the resampling approaches. RUS, SBC, and CNNTL showed the highest performances, with significantly higher Gmean than the original dataset in all the classifiers except NB. Moreover, RUS indicated significantly higher performances than NCL and OSS (Table 4 and Supplementary Table 8).
- *Balanced Accuracy* showed very similar patterns with respect to Gmean, denoting differences for all the classifiers. According to Shaffer's *post-hoc* analysis, CNNTL, RUS, and SBC perform significantly better than the original dataset and the NCL and OSS resampling approaches, being RUS the best algorithms (as shown in Table 4 and Supplementary Table 9).

**Table 4:** Friedman's and *post-hoc* Shaffer's test for the *undersampling* techniques applied to the four performance measures: AUC, Fm, Gmean, and BalACC.

**Undersampling**		***DT***	***DA***	***LR***	***NB***	***SVM***	***KNN***	***EnsBO***	***EnsBA***	***EnsDA***	***EnsKNN***
Original vs. *CNNTL*	AUC	**–**				**–**					
	Fm	**–**		**–**		**–**	**–**	**–**		**–**	**–**
	Gmean	**–**	**–**	**–**		**–**	**–**	**–**	**–**	**–**	**–**
	BalACC	**–**	**–**	**–**	**–**	**–**	**–**	**–**		**–**	**–**
Original vs. *NCL*	AUC	**–**									
	Fm	**–**						**–**	**–**	**–**	
	Gmean										
	BalACC										
Original vs. *OSS*	AUC	**–**									
	Fm	**–**				**–**	**–**				**–**
	Gmean										
	BalACC										
Original vs. *RUS*	AUC	**–**				**–**	**–**				**–**
	Fm	**–**		**–**		**–**	**–**	**–**	**–**	**–**	**–**
	Gmean	**–**	**–**	**–**		**–**	**–**	**–**	**–**	**–**	**–**
	BalACC	**–**	**–**	**–**		**–**	**–**	**–**	**–**	**–**	**–**
Original vs. *SBC*	AUC	**–**				**–**					
	Fm					**–**	**–**				**–**
	Gmean	**–**		**–**		**–**	**–**	**–**	**–**	**–**	**–**
	BalACC	**–**		**–**		**–**	**–**	**–**	**–**	**–**	**–**
*CNNTL* vs. *NCL*	AUC					+			**–**		**–**
	Fm					+	+				**–**
	Gmean			+		+	+	+		+	
	BalACC			+		+	+				
*CNNTL* vs. *OSS*	AUC										
	Fm										
	Gmean				+	+	+	+		+	
	BalACC		+		+	+	+	+		+	
*CNNTL* vs. *RUS*	AUC								**–**		**–**
	Fm										
	Gmean										
	BalACC										
*CNNTL* vs. *SBC*	AUC								**–**		**–**
	Fm							+			
	Gmean										
	BalACC										
*NCL* vs. *OSS*	AUC								+		+
	Fm										
	Gmean										
	BalACC										
*NCL* vs. *RUS*	AUC					**–**					
	Fm					**–**	**–**				**–**
	Gmean	**–**		**–**		**–**	**–**	**–**	**–**	**–**	**–**
	BalACC	**–**		**–**		**–**	**–**	**–**	**–**	**–**	**–**
*NCL* vs. *SBC*	AUC										
	Fm										
	Gmean										
	BalACC			**–**							
*OSS* vs. *RUS*	AUC								**–**		**–**
	Fm										
	Gmean	**–**	**–**	**–**		**–**	**–**	**–**	**–**	**–**	**–**
	BalACC	**–**	**–**	**–**		**–**	**–**	**–**	**–**	**–**	**–**
*OSS* vs. *SBC*	AUC								**–**		**–**
	Fm										
	Gmean										
	BalACC										
*RUS* vs. *SBC*	AUC										
	Fm			+							
	Gmean										
	BalACC										

*The 10 columns refer to the 10 classifiers models. The comparisons showing significant results are indicated with a “–” sign when the first algorithm (of the two compared in each row) was lower or with a “+” sign when it was higher than the second one. The rows without significant differences are not reported. Complete results with the p-values can be found in Supplementary Tables 3–6*.

### Ensemble Methods for Imbalanced Domain

To compare the ensemble methods, we considered the two indices Gmean and BalACC since they have been shown to better capture the differences among the algorithms, as reported in the previous section.

**Figure 4:**
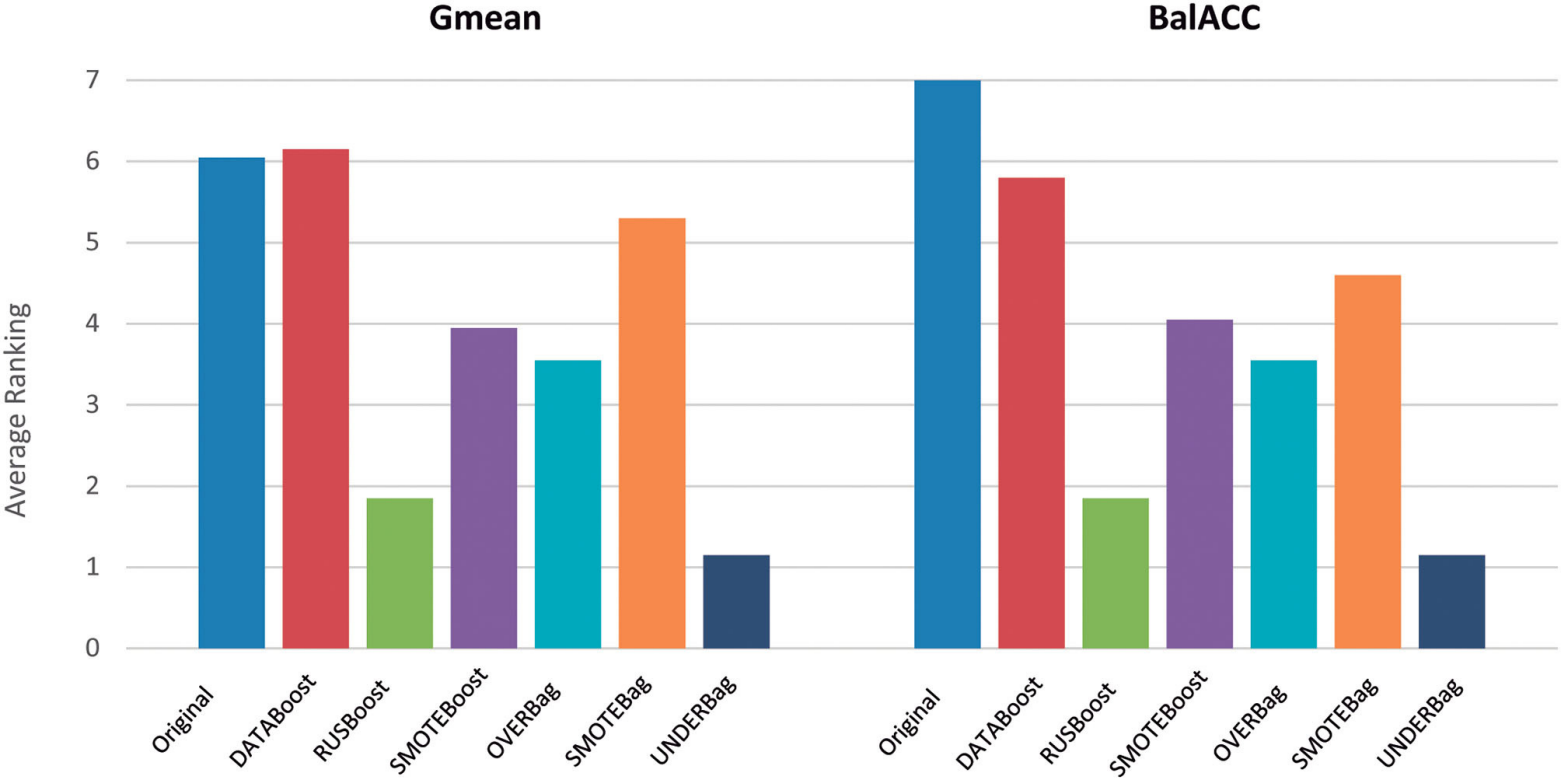
Comparison of performances among the original dataset (blue bars) and six modified ensemble approaches for the imbalanced domain, in terms of the ranking (*y*-axis) of Gmean and BalACC. Lower-ranking values indicate better performances. As shown in Table 4, for the results of statistical comparisons.

Figure 4 shows the average ranking value for each of the proposed ensemble approaches, for both Gmean and BalACC. Corresponding results according to *post-hoc* Shaffer's test, comparing the seven approaches (original dataset and six ensembles) can be found in Table 5. According to Friedman's test, both the measures indicated significant differences among these techniques (Gmean: *p* < 0.00001; BalACC: *p* < 0.00001). A *post-hoc* analysis pointed out that DATABoost and SMOTEBag did not improve the performances with respect to the original dataset, and that SMOTEBoost, OVERBag showed higher BalACC than the original data but no differences in terms of Gmean. On the contrary, RUSBoost and UNDERBag showed significantly better performances than all the other algorithms, being UNDERBag the best one (Table 5).

**Table 5:** Shaffer's test for the ensemble approaches for the imbalance domain.

**Ensemble**	**AUC**		**Fm**		**Gmean**		**BalACC**	
Original vs. *DATABoost*	–	0.000	–	0.014		1.895		1.285
Original vs. *RUSBoost*		2.344		0.789	–	0.000	–	0.000
Original vs. *SMOTEBoost*		0.165		0.423		0.297	–	0.034
Original vs. *OVERBag*		0.555		1.285		0.106	–	0.005
Original vs. *SMOTEBag*	–	0.040	–	0.006		1.895		0.143
Original vs. *UNDERBag*		2.344	–	0.000	–	0.000	–	0.000
*DATABoost* vs. *RUSBoost*	+	0.000		0.789	–	0.000	–	0.001
*DATABoost* vs. *SMOTEBoost*		0.555		1.285		0.251		0.491
*DATABoost* vs. *OVERBag*		0.218		0.372		0.078		0.199
*DATABoost* vs. *SMOTEBag*		1.499		1.814		1.895		1.285
*DATABoost* vs. *UNDERBag*	+	0.000		0.298	–	0.000	–	0.000
*RUSBoost* vs. *SMOTEBoost*		0.165		1.814		0.297		0.205
*RUSBoost* vs. *OVERBag*		0.555		1.814		0.549		0.549
*RUSBoost* vs. *SMOTEBag*	+	0.040		0.701	+	0.005	+	0.049
*RUSBoost* vs. *UNDERBag*		2.344	–	0.002		1.895		1.406
*SMOTEBoost* vs. *OVERBag*		2.344		1.516		1.895		1.406
*SMOTEBoost* vs. *SMOTEBag*		2.344		1.031		0.974		1.406
*SMOTEBoost* vs. *UNDERBag*	–	0.024	–	0.006		0.056	–	0.034
*OVERBag* vs. *SMOTEBag*		1.663		0.298		0.491		1.285
*OVERBag* vs. *UNDERBag*		0.091	–	0.000		0.143		0.143
*SMOTEBag* vs. *UNDERBag*	–	0.003		0.423	–	0.000	–	0.005

*Red color indicates the p-values with significant differences according to Shaffer's post-hoc (p <0.05); the sign “–” (respectively “+”) indicates that the first algorithm has a lower (higher) value than the second one*.

**Figure 5:**
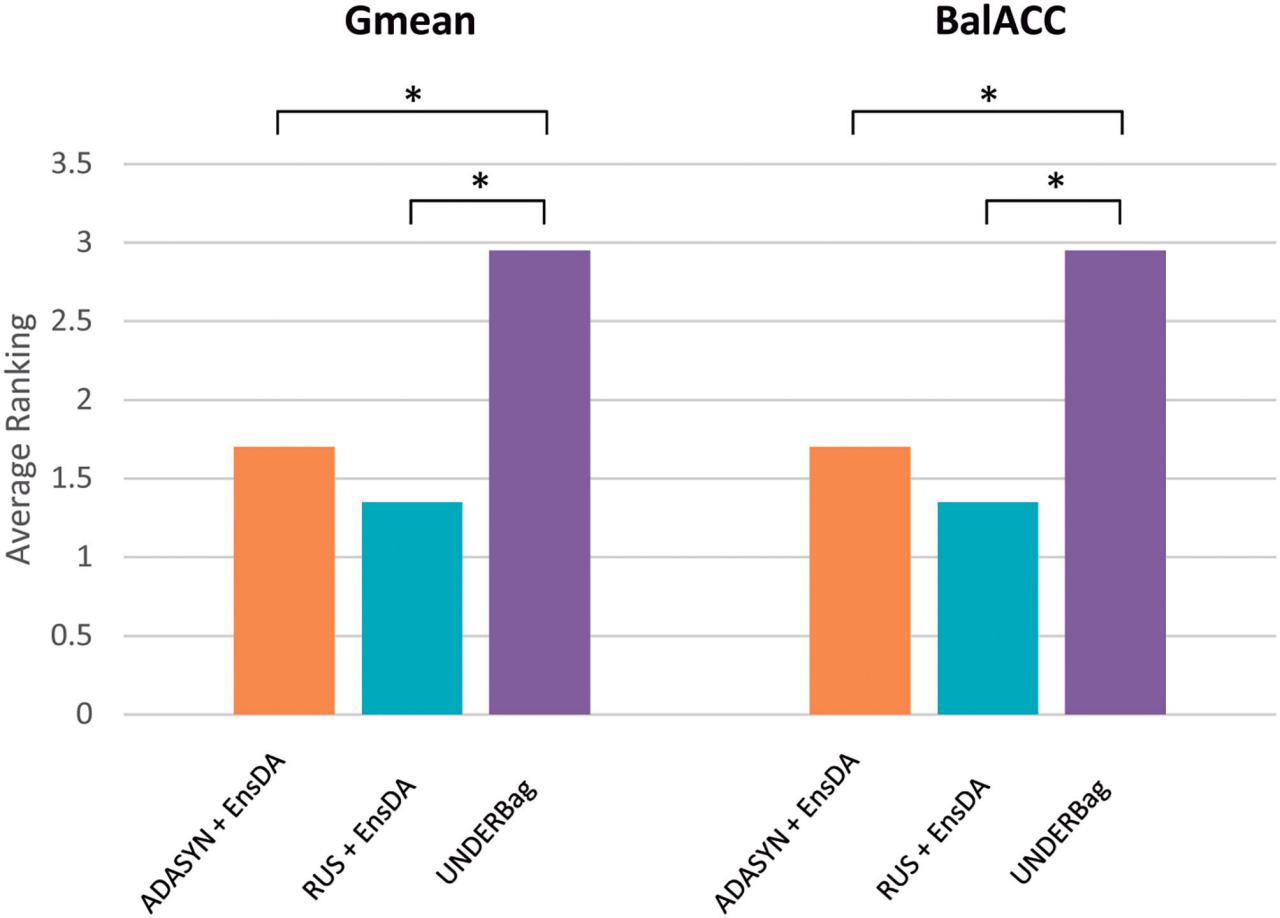
A final comparison of performances among the best approach for each of the resampling families considered: (1) Oversampling: ADASYN + EnsDA [Adaptive Synthetic Sampling (ADASYN) combined with “classical” ensemble approach EnsembleDA; orange bars]; (2) Undersampling: RUS + EnsDA [Random Undersampling (RUS) combined with “classical” ensemble approach *EnsembleDA*—blue bars]; (3) Specific ensemble learner for imbalanced domain: UNDERBagging (violet bars). *Y*-axis represents ranking values, for both the Gmean and BalACC. The lower-ranking values indicate better performances. *indicates significant differences according to Shaffer's *post-hoc* analysis.

Since in the previous section we used classical ensemble classifiers combined with a rebalancing pre-processing step, we also compared the one with better performances (EnsDA, after ADASYN and RUS resampling) with the best algorithm of the modified ensemble family UNDERBag. Interestingly, EnsDA, with both ADASYN and RUS pre-processing, showed significantly higher Gmean and BalACC than the UNDER_Ba approach (*p* < 0.00519 for ADASYN+Ens_DA vs. UNDERBag, and *p* < 0.00104 for RUS+Ens_DA vs. UNDERBag, for both Gmean and BalACC). Figure 5 represents the comparison among these three methods, expressed in terms of ranking values.

### Sensitivity and Specificity

**Figure 6:**
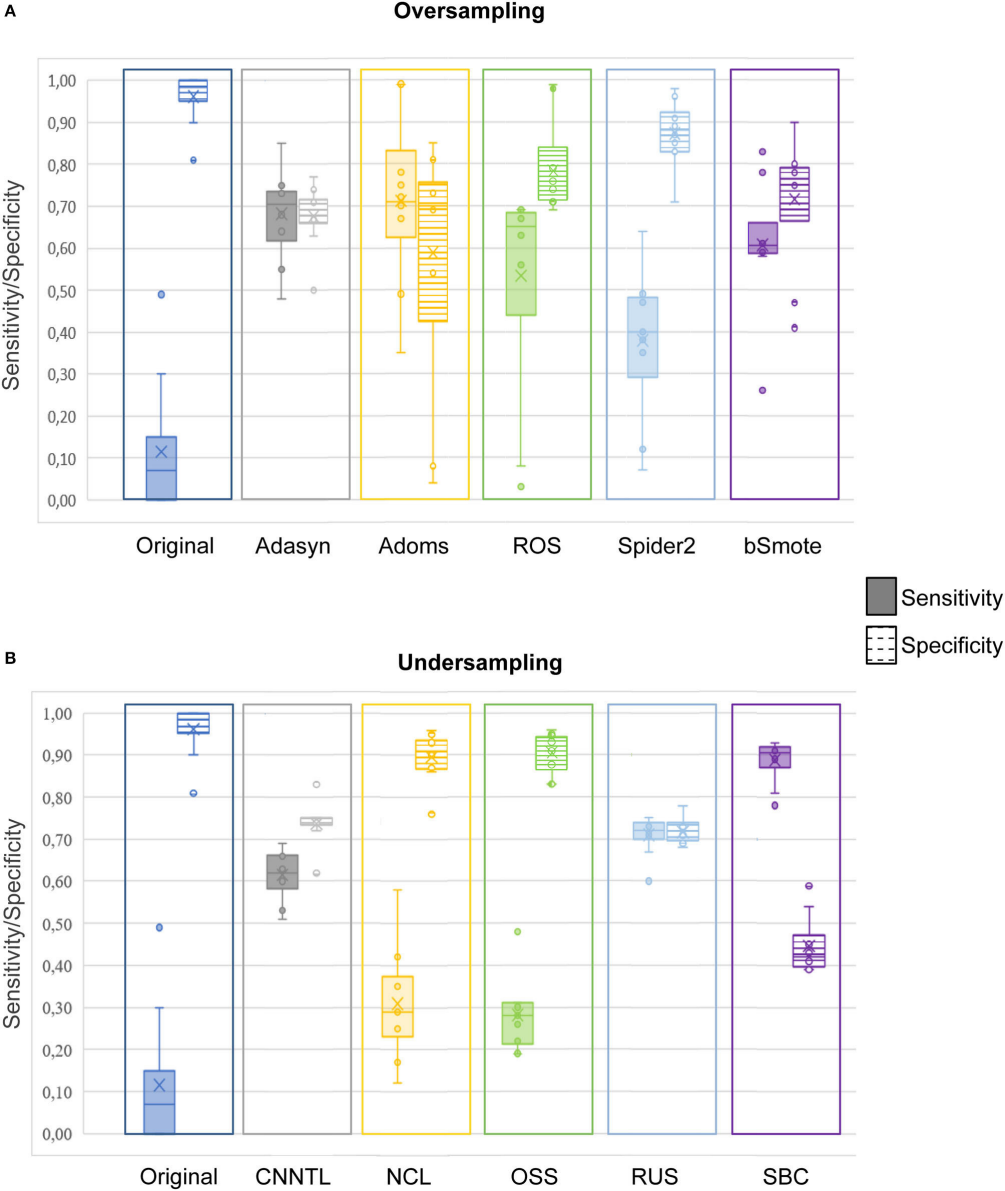
Boxplot representing the values of sensitivity (full-color bars) and specificity (horizontal lines bars) for the original dataset and the five oversampling **(A)** and undersampling approaches **(B)**. Each box represents the variability among the 10 classification models. The middle line indicates the median value; upper and lower limits of the box indicate the first and third quartile; external points indicate outliers; x indicates the mean values. The corresponding values can be found in Table 6.

To clarify the effective use of the proposed approach to EZ identification, we reported sensitivity and specificity for the different techniques tested in the study. Since ensemble approaches showed significantly lower performances than resampling in terms of performances metrics (as indicated in the previous paragraph), only the sensitivity and specificity of the latter were further analyzed. Figure 6 shows the boxplots indicating the values of sensitivity (full-color boxes) and specificity (horizontal lines boxes) for the original dataset compared with the five oversampling (Figure 6A) and the five undersampling approaches (Figure 6B). Each box represents the variability among the 10 classification models. All sensitivity and specificity values are reported in Table 6. Such results confirmed the main evidence obtained by the other performance metrics: (i) original data were not able to provide a good classification, since all the models tended to classify the whole set of leads as non-EZ (sensitivity ≈ 0; specificity ≈ 1), confirming the biased classification toward the majority non-EZ class; (ii) oversampling improved classification performances, especially in terms of sensitivity. The Adasyn method provided the highest combination of both values (sensitivity and specificity >0.7) and the lowest variability of performances among the classification models. The ADOMS method showed average performances comparable with ADASYN, but much more variability with respect to the model choice. The SPIDER method was the least effective approach to improve the performances; (iii) Some undersampling approaches improved the classification performances, but with a strong variability among the different methods. NCL and OSS show results comparable to the original dataset. The RUS method provided the highest values of both sensitivity and specificity, comparable with the ADASYN approach. Interestingly, the SBC showed the highest sensibility values (≈0.9), even if associated with a less balanced specificity.

**Table 6:** The Sensitivity (Sens) and Specificity (Spec) values for oversampling and undersampling techniques.

	**Oversampling**											
	**Orig**		**Adasyn**		**Adoms**		**ROS**		**Spider2**		**bSmote**	
	***Sens***	***Spec***	***Sens***	***Spec***	***Sens***	***Spec***	***Sens***	***Spec***	***Sens***	***Spec***	***Sens***	***Spec***
DT	0.00	1.00	0.75	0.63	0.78	0.54	0.63	0.76	0.49	0.83	0.59	0.75
DA	0.30	0.90	0.64	0.74	0.49	0.81	0.56	0.79	0.47	0.83	0.58	0.78
LR	0.08	0.98	0.73	0.71	0.72	0.74	0.68	0.74	0.40	0.89	0.61	0.79
NB	0.49	0.81	0.68	0.68	0.67	0.69	0.67	0.69	0.64	0.71	0.62	0.73
SVM	0.00	1.00	0.73	0.68	0.75	0.70	0.69	0.72	0.35	0.91	0.60	0.80
KNN	0.00	1.00	0.68	0.67	0.68	0.70	0.63	0.71	0.40	0.87	0.59	0.75
EnsBO	0.06	0.99	0.85	0.50	0.99	0.08	0.67	0.72	0.48	0.85	0.83	0.47
EnsBA	0.10	0.97	0.55	0.68	0.99	0.04	0.08	0.98	0.12	0.96	0.78	0.41
EnsDA	0.09	0.98	0.73	0.71	0.70	0.73	0.69	0.74	0.38	0.90	0.61	0.78
EnsKNN	0.03	0.99	0.48	0.77	0.35	0.85	0.03	0.99	0.07	0.98	0.26	0.90
**Mean**	**0.12**	**0.96**	**0.68**	**0.68**	**0.71**	**0.59**	**0.53**	**0.78**	**0.38**	**0.87**	**0.61**	**0.72**
**St. Dev**	***0.16***	***0.06***	***0.11***	***0.07***	***0.20***	***0.29***	***0.26***	***0.11***	***0.17***	***0.08***	***0.15***	***0.15***
	**Undersampling**											
	**Orig**		**CNNTL**		**NCL**		**OSS**		**RUS**		**SBC**	
	***Sens***	***Spec***	***Sens***	***Spec***	***Sens***	***Spec***	***Sens***	***Spec***	***Sens***	***Spec***	***Sens***	***Spec***
DT	0.00	1.00	0.61	0.74	0.35	0.87	0.28	0.91	0.71	0.71	0.92	0.39
DA	0.30	0.90	0.51	0.83	0.42	0.86	0.30	0.91	0.60	0.78	0.78	0.59
LR	0.08	0.98	0.63	0.75	0.26	0.91	0.28	0.92	0.71	0.74	0.91	0.45
NB	0.49	0.81	0.61	0.74	0.58	0.76	0.48	0.83	0.67	0.69	0.81	0.54
SVM	0.00	1.00	0.69	0.72	0.12	0.96	0.19	0.96	0.75	0.70	0.92	0.41
KNN	0.00	1.00	0.67	0.74	0.17	0.95	0.19	0.95	0.74	0.70	0.93	0.39
EnsBO	0.06	0.99	0.64	0.75	0.29	0.91	0.26	0.93	0.73	0.70	0.92	0.41
EnsBA	0.10	0.97	0.53	0.74	0.36	0.90	0.31	0.88	0.74	0.73	0.90	0.43
EnsDA	0.09	0.98	0.66	0.74	0.29	0.91	0.22	0.94	0.71	0.74	0.90	0.45
EnsKNN	0.03	0.99	0.60	0.62	0.25	0.93	0.31	0.83	0.74	0.68	0.89	0.40
**Mean**	**0.12**	**0.96**	**0.62**	**0.74**	**0.31**	**0.90**	**0.28**	**0.91**	**0.71**	**0.72**	**0.89**	**0.45**
**St. Dev**	***0.16***	***0.06***	***0.06***	***0.05***	***0.13***	***0.06***	***0.08***	***0.05***	***0.05***	***0.03***	***0.05***	***0.07***

*Single values for each of the 10 classifier models, as well as mean and standard deviation (St.Dev.) are indicated*.

**Figure 7:**
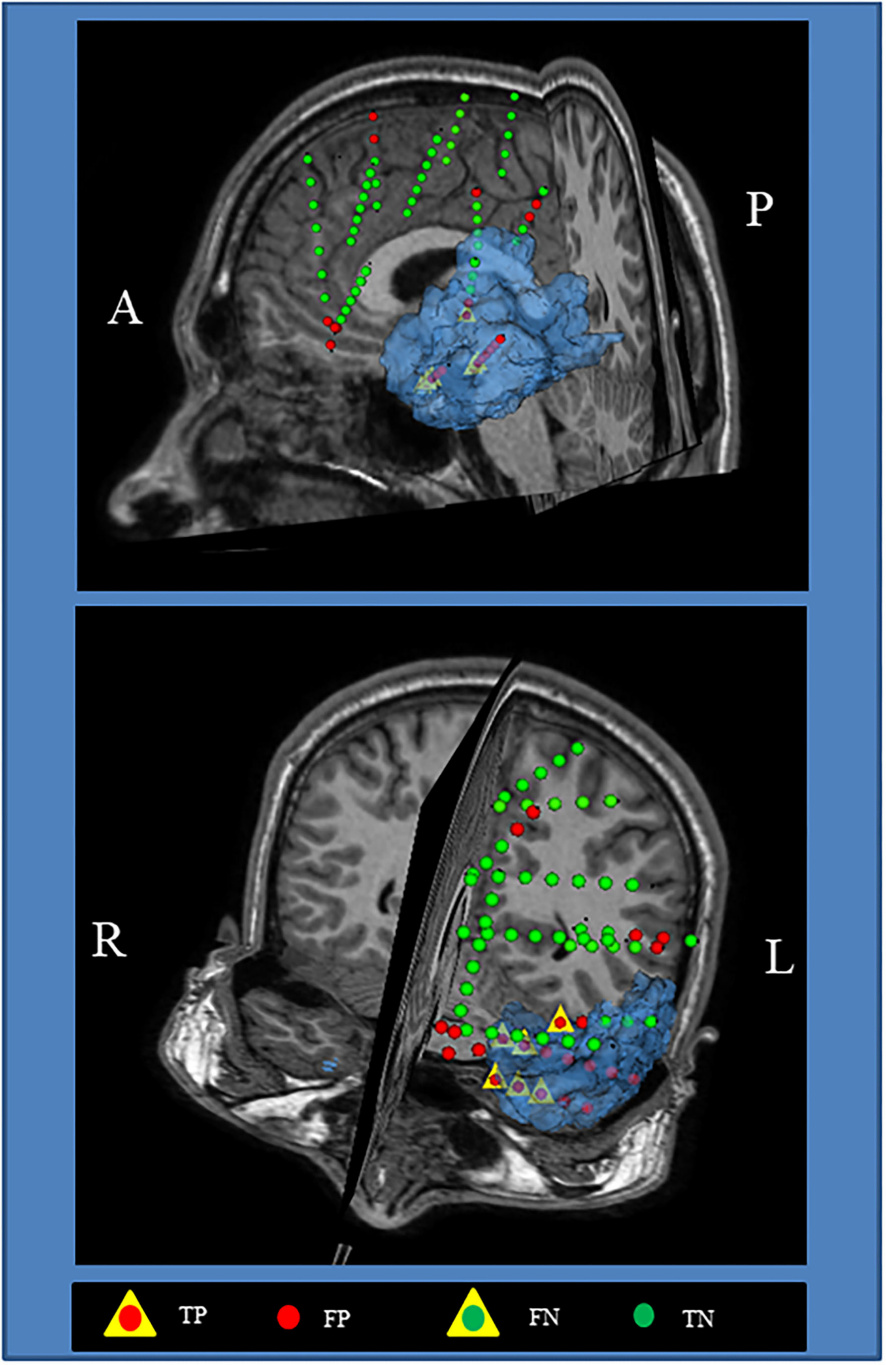
Visualization of the surgical 3D scene for a representative patient (pt2). The Blue area indicates the final resected zone. Red and green dot points indicate leads classified as epileptogenic zone (EZ) A, anterior; L, left; P, posterior; R, right.

Figure 7 shows the visualization of the surgical 3D scene for a representative patient (pt2), such as an indication of the resected zone (blue area), true EZ and non-EZ leads, and the EZ and non-EZ classification provided by the RUS + EnsDA method.

## Discussion

Machine learning approaches are being increasingly applied to the field of epilepsy, and specifically in the different datasets from neurophysiological recordings (Abbasi and Goldenholz, 2019). In this context, it is quite common to cope with the imbalanced datasets characterized by uneven distribution between majority and minority classes, which can lead to worse classification performances.

This is the case of the EZ localization in the pre-surgical planning to achieve seizure freedom after surgical resection of the EZ. One assessed clinical practice is the exploration through intracranial EEG recordings (SEEG) (Cardinale et al., 2019) combined with the visual analysis and advanced signal processing methods able to extract quantitative indexes to support the correct EZ localization (Bartolomei et al., 2017).

Intentionally, to sample a wide region of the epileptic brain, the explored brain regions are much wider than the true EZ, thus resulting in an imbalanced class distribution between EZ and non-EZ contacts, with the EZ being the most important class to be correctly identified to reduce or remove seizures, being the minority class. This led the classifier to be biased toward the majority (non-EZ) class.

Starting from the evidence that network analysis of interictal SEEG recordings could be very useful in support of the EZ localization (Varotto et al., 2012; Vlachos et al., 2017; Lagarde et al., 2018), in this study we demonstrated that the combination of supervised machine learning with appropriate data resampling approach can strongly improve its potential. For this reason, the idea of applying resampling techniques in the field of EZ localization should be taken into consideration.

At present, no study investigated the effect of imbalance domains on the performance of EZ localization methodologies. The previous studies demonstrated that the application of rebalancing techniques could strongly improve the classification of EEG signals for epilepsy diagnosis (Haldar et al., 2019; Kaur et al., 2020) and automatic seizure detection (Cosgun et al., 2019; Romaissa et al., 2019; Masum et al., 2020). However, in most of them, the well-known and assessed resampling techniques belonging to the SMOTE family were applied, and systematic comparison with other possible approaches was missing.

In this study, we compared five oversampling and five undersampling procedures and tested the resulting rebalanced datasets with 10 different machine learning classifiers. Moreover, we also tested six specific ensemble methods properly modified for imbalanced domain and belonging to data variation-based ensemble.

Our study focuses on identifying the best resampling and classification approach to support the classification of brain regions as EZ or non-EZ, using the indexes derived from connectivity and graph-theory analysis of interictal SEEG recording as features. The selection of the nine graph-theory-based indexes used as input features of the classifiers was based on the preliminary analysis we performed, showing that the combination of these indexes was the most appropriate to achieve the best EZ classification. In the contest of EZ localization, despite the early application of several other signal processing approaches for feature extraction, such as working in the frequency domain or by non-linear analysis, network analysis started only recently to be employed based on the evidence that focal epilepsy is a network disease. However, most of these recent network studies normally focus only on the connectivity analysis that is rarely combined with the pre-processing approaches, due to the huge amount of data to be processed. For this reason, in this study, we mainly focused on presenting pre-processing, in combination with a few of such feature extraction and connectivity measures in the literature, to provide evidence of and support for a proper pre-processing method in this context.

Regarding oversampling, all five approaches reported improved performances with respect to the original dataset. The differences among the five oversampling approaches varied according to the considered classifiers.

Adaptive Synthetic Sampling resulted to be the most robust approach among the classifiers. ADOMS was the less robust and most sensitive to the choice of classifier, being comparable or even slightly better than ADASYN in LR, SVM, KNN, EnsDA, and EnsKNN, while as bad as the original dataset in DA, EnsBO, and EnsBA. SPIDER was the least effective, with performances significantly worse than the other approaches and comparable with the original dataset for some classifiers, especially the classical ensemble family.

Regarding undersampling, all the approaches appeared to be less influenced by the classifier choice than the oversampling. Two of the proposed methods, NCL and OSS, did not improve the classification performances with respect to the original data. The other approaches were significantly better than original data, with RUS, the simplest of the proposed methods, being the best one.

Interestingly RUS showed higher, even not significant, performances than the best oversampling approach, ADASYN.

The resampling technique is not the only family to cope with the imbalanced domain. A wide number of approaches exist to deal with this problem, which can be mainly categorized as data-level or algorithmic-level approaches (López et al., 2013). Rebalancing belongs to the data-level approaches, in which data are pre-processed before the classification (Lee, 2014). On the contrary, in the algorithmic-level ones, the classification algorithm is modified to deal with the imbalanced nature (Barandela et al., 2003a). The cost-sensitive approaches combine both the data and algorithmic levels, by assigning different misclassification costs for the two classes and modify the classification algorithm to minimize the higher misclassification cost (Domingos, 1999; Zhou and Liu, 2006; Sun et al., 2007).

The main limitation of cost-sensitive approaches is the need of defining the correct misclassification costs for the two classes, which may not be so clear in many clinical problems, as in our case.

In this paper, we focused on the rebalancing techniques since they can be quite easily implemented, and are independent of the underlying classifiers, which can be an advantage in problems where the selection of the most appropriate classifier is not clear (Batista et al., 2004; Batuwita and Palade, 2010).

In addition, several modifications of ensemble methods for the imbalanced domain have been proposed (Rokach, 2010), both working at data-level approach, through the data pre-processing before each step of the ensemble classification (Breiman, 1996; Freund and Schapire, 1997; Kuncheva, 2014), or with algorithmic-level cost-sensitive modification (Sun et al., 2007).

As part of the data-level approaches, we considered and tested, in this study, six different data-level ensemble algorithms. As reported in a previous study (Galar et al., 2012), we found that the simplest algorithms, UNDERBag and RUSBoost emerged as the best ensemble methods, while offering lower computation costs.

Interestingly, when compared these results with those obtained by a standard single-step resampling approach combined with a classical ensemble algorithm, we found significantly higher performances in the latter family, in particular for the combination (ADASYN + EnsDA and RUS + EnsDA).

This highlights again that the simplest algorithms guarantee high performances, and that their very low computational complexity can be a strong advantage toward routine clinical applications.

It is important to notice that the performances of the different resampling techniques are strongly influenced by the choice of the classifier. This highlights that the selection of the resampling approach for a specific dataset should always take into consideration the choice of the classifier.

Regarding the measure to assess and compare the performances, in this study we applied four measures considered most appropriate to deal with imbalanced classification: AUC, Fm, Gmean, and BalACC (Bekkar et al., 2013). Several studies already highlighted that the choice of the proper evaluation measures for model assessment is one of the most complex issues faced in the imbalanced data learning context and how the application of more standard measures, such as accuracy, could lead to erroneous interpretations and biased classification (Weiss, 2004).

These four measures provided complementary results and to properly evaluate the performances of different approaches, it is important to take into account the combination of them, especially considering which aspect is more important in the specific problem we are facing. Particularly, in this case, we noticed that AUC and Fm did not completely capture differences in the model performances. On the other side, as already described in another paper (Luque et al., 2019), Gmean and BalACC appear to be good performance metrics when the main focus is to maximize sensitivity, without losing too much specificity.

## Data Availability Statement

Data are available from the corresponding authors upon request. Requests to access these datasets should be directed to giulia.varotto@istituto-besta.it.

## Ethics Statement

The study was approved by the Ethics Committee of the Fondazione IRCCS Istituto Neurologico Carlo Besta of Milan and was carried out in accordance with the ethical standards laid down in the 1964 Declaration of Helsinki and its later amendments. All of the subjects gave their written informed consent before being included in the study.

## Author Contributions

GV: designed and conceptualized the study, analyzed and interpreted the data, and drafted the manuscript for intellectual content. GS: contributed to design the study, analyzed the data, and contributed to draft and revise the manuscript. LT and FG: major role in the acquisition of data and contributed to revise the manuscript. SF and FP: interpreted the data and contributed to draft and revise the manuscript. All authors contributed to the article and approved the submitted version.

## Funding

This study was funded by the DESIRE (Strategies for Innovative Research to Improve Diagnosis, Prevention, and Treatment in children with difficult to treat epilepsy), an FP7 funded project (Grant Agreement No. 602531), from the European Commission, and the Grants Nos. RF-2011-02350578 and RF-2010-2319316 from the Italian Ministry of Health.

## Conflict of Interest

The authors declare that the research was conducted in the absence of any commercial or financial relationships that could be construed as a potential conflict of interest.

## Publisher's Note

All claims expressed in this article are solely those of the authors and do not necessarily represent those of their affiliated organizations, or those of the publisher, the editors and the reviewers. Any product that may be evaluated in this article, or claim that may be made by its manufacturer, is not guaranteed or endorsed by the publisher.

## Supplementary Material

Supplementary Figure 1 A subset of stereo-electroencephalography (SEEG) traces recorded from pt2, and corresponding adjacency matrices for the first 3 of the 36 epochs analyzed.

Supplementary Table 1 Description of the set of graph-theory based centrality measures used in this study.

Supplementary Table 2 Friedman and *post-hoc* Shaffer test for the *oversampling* techniques with *AUC* measure. Shaffer *post-hoc* comparisons have been indicated only when Friedman test resulted significant (*p*-values in the first line). Red color indicates *p*-values with significant differences according to shaffer *post-hoc* (*p* < 0.05); “–” (respectively “+”) indicates that the first algorithm has lower (higher) value than the second one.

Supplementary Table 3 Friedman and *post-hoc* Shaffer test for the *oversampling* techniques with *Fm* measure. Shaffer *post-hoc* comparisons have been indicated only when Friedman test resulted significant (*p*-values in the first line). Red color indicates *p*-values with significant differences according to shaffer *post-hoc* (*p* < 0.05); “–” (respectively “+”) indicates that the first algorithm has lower (higher) value than the second one.

Supplementary Table 4 Friedman and *post-hoc* Shaffer test for the *oversampling* techniques with *Gmean* measure. Shaffer *post-hoc* comparisons have been indicated only when Friedman test resulted significant (*p*-values in the first line). Red color indicates *p*-values with significant differences according to shaffer *post-hoc* (*p* < 0.05); “–” (respectively “+”) indicates that the first algorithm has lower (higher) value than the second one.

Supplementary Table 5 Friedman and *post-hoc* Shaffer test for the *oversampling* techniques with *BalACC* measure. Shaffer *post-hoc* comparisons have been indicated only when Friedman test resulted significant (*p*-values in the first line). Red color indicates *p*-values with significant differences according to shaffer *post-hoc* (*p* < 0.05); “–” (respectively “+”) indicates that the first algorithm has lower (higher) value than the second one.

Supplementary Table 6 Friedman and *post-hoc* Shaffer test for the *oversampling* techniques with *AUC* measure. Shaffer *post-hoc* comparisons have been indicated only when Friedman test resulted significant (*p*-values in the first line). Red color indicates *p*-values with significant differences according to shaffer *post-hoc* (*p* < 0.05); “–” (respectively “+”) indicates that the first algorithm has lower (higher) value than the second one.

Supplementary Table 7 Friedman and *post-hoc* Shaffer test for the *undersampling* techniques with *Fm* measure. Shaffer *post-hoc* comparisons have been indicated only when Friedman test resulted significant (*p*-values in the first line). Red color indicates *p*-values with significant differences according to shaffer *post-hoc* (*p* < 0.05); “–” (respectively “+”) indicates that the first algorithm has lower (higher) value than the second one.

Supplementary Table 8 Friedman and *post-hoc* Shaffer test for the *underampling* techniques with *Gmean* measure. Shaffer *post-hoc* comparisons have been indicated only when Friedman test resulted significant (*p*-values in the first line). Red color indicates *p*-values with significant differences according to shaffer *post-hoc* (*p* < 0.05); “–” (respectively “+”) indicates that the first algorithm has lower (higher) value than the second one.

Supplementary Table 9 Friedman and *post-hoc* Shaffer test for the *undersampling* techniques with *BalACC* measure. Shaffer *post-hoc* comparisons have been indicated only when Friedman test resulted significant (*p*-values in the first line). Red color indicates *p*-values with significant differences according to shaffer *post-hoc* (*p* < 0.05); “–” (respectively “+”) indicates that the first algorithm has lower (higher) value than the second one.
