# Supplementary figures and images for: Comparison of Resampling Techniques for Imbalanced Datasets in Machine Learning: Application to Epileptogenic Zone Localization From Interictal Intracranial EEG Recordings in Patients With Focal Epilepsy
Source: Front Neuroinform. 2021 Nov 19;15:715421. doi: 10.3389/fninf.2021.715421 (PMC8641296; doi:10.3389/fninf.2021.715421)

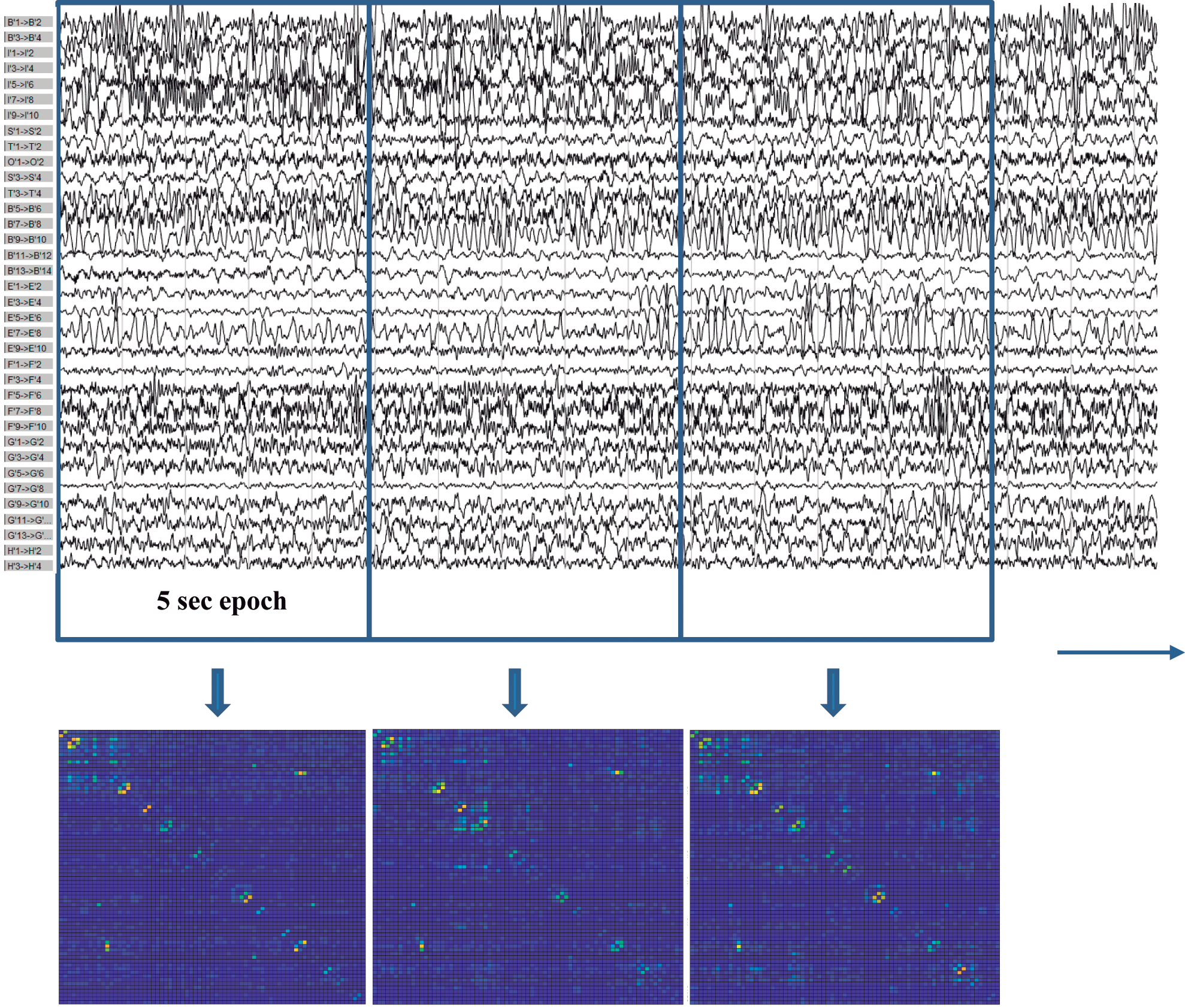

Supplement: Supplementary Figure 1 — A subset of stereo-electroencephalography (SEEG) traces recorded from pt2, and corresponding adjacency matrices for the first 3 of the 36 epochs analyzed. [file Image_1.TIF]
