# Supplementary material for: Comparison of Resampling Techniques for Imbalanced Datasets in Machine Learning: Application to Epileptogenic Zone Localization From Interictal Intracranial EEG Recordings in Patients With Focal Epilepsy
Source: Front Neuroinform. 2021 Nov 19;15:715421. doi: 10.3389/fninf.2021.715421 (PMC8641296; doi:10.3389/fninf.2021.715421)
Supplement: Supplementary Table 1 — Description of the set of graph-theory based centrality measures used in this study. [file Table_1.DOCX]

**Supplementary material**

### Nonlinear analytical method used to generate the adjacency matrices.

### Considering the interictal intervals, we extracted the *non-linear correlation coefficient*. The latter has been introduced in the field of EEG analysis by Lopes da Silva et al. (1989), as a non-parametric method for characterizing the dependency of a signal *Y* on a signal *X*, independently of the type of relation between the two signals.

In short, the dependency between considered signals is quantified by a normalized non-linear correlation coefficient *h^2^_XY_* given by

${h^{2}}_{XY}\left( \tau\right)=1-\frac{VAR[Y(t+\tau)/X(t)]}{VAR[Y(t+\tau)]}$ (S1)

where

$VAR\left[ Y\left( t+\tau\right)/X(t) \right]\hat{=}\arg min\left( E\left[ Y\left( t+\tau\right)-h\left( X\left( t \right) \right) \right]^{2} \right)$ (S2)

and *h*is a non-linear fitting curve which approximates the statistical relationship between *X*and *Y*.

**Supplementary Table 1.** Description of the set of graph-theory based centrality measures used in this study.

| **Centrality index** | **Expression** | **Description** |
| --- | --- | --- |
| Outdegree c. | $C_{OD}\left( i \right)= \sum_{j\neq i} sgn (w_{ij})$  where $sgn(x)$ is the sign function. | Number of edges outcoming from the considered node |
| Indegree c. | $C_{ID}\left( i \right)= \sum_{j\neq i} {sgn (w}_{ji})$ | Number of edges incoming to the considered node |
| Outstrength c. | $C_{OS}\left( i \right)= \sum_{j\neq i} w_{ij}$ | Sum of the edge weights outcoming from the considered node |
| Instrength c. | $C_{IS}\left( i \right)= \sum_{j\neq i} w_{ji}$ | Sum of the edge weights incoming to the considered node |
| Betweenness c. | $C_{B}\left( i \right)= \frac{1}{(N-1) (N-2)} \sum_{h\neq i h\neq j j\neq i} \frac{\rho_{hj}(i)}{\rho_{hj}}$  where$\rho_{hj}(i)$ is the number of shortest paths between j and k that pass through *i*, $\rho_{hj}$ is the number of shortest paths between *j* and *k*, and $(N-1)(N-2)$ is the number of node pairs that does not include node *i*. | It measures of how often the considered node appears on a shortest path between two nodes in the graph. |
| Outcloseness c. | $C_{OC}\left( i \right)=\frac{1}{\sum_{j=1}^{N} d_{ij}}$  Where $d_{ij}$ is the topological distance from node i to node j | It measures the inverse sum of the distance of the shortest paths from the considered node to all other nodes in the graph |
| Incloseness c. | $C_{IC}\left( i \right)=\frac{1}{\sum_{j=1}^{N} d_{ji}}$  Where $d_{ji}$ is the distance from all the j nodes to the node i. | It measures the inverse sum of the distance of the shortest paths from all nodes of the graph to the considered node. |
| Page Rank c. | $C_{PR}\left( i \right)={D (D-\alpha A)}^{-1}\boldsymbol{1}$,  where **1** is a column vector of ones of length *N_L_* , *D* is the diagonal matrix of node outdegrees, and α is a free parameter which weights the contribution of network topology to the centrality score. | This measure scales the contributions that the neighbors of node *i* make to its centrality by the degree of those neighbors, thereby accounting for any potential bias associated with links to highly connected nodes. |
| Eigenvector c. | $C_{E} (i) =\frac{1}{\lambda_{1}}\sum_{j=1}^{N} A_{ij}C_{E}(j)$  Where A is the adjacency matrix and λ_1_ its largest eigenvalue. | Recursive measure that considers both the degree of the considered node and the degree of its neighbors. |

**Supplementary Table 2**

Friedman and post-hoc Shaffer test for the *oversampling* techniques with *AUC* measure. Shaffer post-hoc comparisons have been indicated only when Friedman test resulted significant (p-values in the first line). Red colour indicates p-values with significant differences according to shaffer post-hoc (p<0.05); “-” (respectively “+”) indicates that the first algorithm has lower (higher) value than the second one.

|  |  | **DT** | | **DA** | | **LR** | | **NB** | | **SVM** | | **KNN** | | **EnsBO** | | **EnsBA** | | **EnsDA** | | **EnsKNN** | |
| --- | --- | --- | --- | --- | --- | --- | --- | --- | --- | --- | --- | --- | --- | --- | --- | --- | --- | --- | --- | --- | --- |
| **FRIEDMAN TEST** | |  | 0.000 |  |  |  |  |  |  |  | 0.003 |  |  |  | 0.002 |  | 0.000 |  |  |  | 0.000 |
| **SHAFFER post-hoc** | Original vs. *ADASYN* | **-** | 0.000 |  |  |  |  |  |  | **-** | 0.013 |  |  | - | 0.019 |  | 1.000 |  |  | - | 0.034 |
|  | Original vs. *ADOMS* |  | 0.198 |  |  |  |  |  |  | - | 0.010 |  |  |  | 1.000 | + | 0.001 |  |  | - | 0.000 |
|  | Original vs. *ROS* | **-** | 0.005 |  |  |  |  |  |  | - | 0.010 |  |  |  | 1.000 |  | 1.000 |  |  |  | 0.086 |
|  | Original vs. *SPIDER* | **-** | 0.000 |  |  |  |  |  |  |  | 0.730 |  |  |  | 1.000 |  | 1.000 |  |  |  | 1.000 |
|  | Original vs. *bSMOTE* |  | 0.060 |  |  |  |  |  |  | **-** | 0.028 |  |  |  | 1.000 |  | 1.000 |  |  | - | 0.000 |
|  | *ADASYN* vs. *ADOMS* |  | 0.270 |  |  |  |  |  |  |  | 1.000 |  |  | + | 0.015 |  | 0.072 |  |  |  | 1.000 |
|  | *ADASYN* vs. *ROS* |  | 1.000 |  |  |  |  |  |  |  | 1.000 |  |  |  | 1.000 |  | 1.000 |  |  |  | 0.086 |
|  | *ADASYN* vs. *SPIDER* |  | 1.000 |  |  |  |  |  |  |  | 1.000 |  |  |  | 1.000 |  | 0.073 |  |  |  | 1.000 |
|  | *ADASYN* vs. *bSMOTE* |  | 0.511 |  |  |  |  |  |  |  | 1.000 |  |  |  | 1.000 |  | 1.000 |  |  |  | 1.000 |
|  | *ADOMS* vs. *ROS* |  | 1.000 |  |  |  |  |  |  |  | 1.000 |  |  | - | 0.000 | - | 0.001 |  |  | + | 0.001 |
|  | *ADOMS* vs. *SPIDER* |  | 0.511 |  |  |  |  |  |  |  | 1.000 |  |  | - | 0.000 | - | 0.000 |  |  |  | 1.000 |
|  | *ADOMS* vs. *bSMOTE* |  | 1.000 |  |  |  |  |  |  |  | 1.000 |  |  |  | 0.060 |  | 0.102 |  |  |  | 1.000 |
|  | *ROS* vs. *SPIDER* |  | 1.000 |  |  |  |  |  |  |  | 1.000 |  |  |  | 1.000 |  | 1.000 |  |  |  | 1.000 |
|  | *ROS* vs. *bSMOTE* |  | 1.000 |  |  |  |  |  |  |  | 1.000 |  |  |  | 1.000 |  | 1.000 |  |  | - | 0.000 |
|  | *SPIDER* vs. *bSMOTE* |  | 0.185 |  |  |  |  |  |  |  | 1.000 |  |  |  | 1.000 |  | 0.073 |  |  |  | 1.000 |

**Supplementary Table 3**

Friedman and post-hoc Shaffer test for the *oversampling* techniques with *Fm* measure. Shaffer post-hoc comparisons have been indicated only when Friedman test resulted significant (p-values in the first line Red colour indicates p-values with significant differences according to shaffer post-hoc (p<0.05); “-” (respectively “+”) indicates that the first algorithm has lower (higher) value than the second one.

|  |  | **DT** | | **DA** | | **LR** | | **NB** | | **SVM** | | **KNN** | | **EnsBO** | | **EnsBA** | | **EnsDA** | | **EnsKNN** | |
| --- | --- | --- | --- | --- | --- | --- | --- | --- | --- | --- | --- | --- | --- | --- | --- | --- | --- | --- | --- | --- | --- |
| **FRIEDMAN TEST** | |  | 0.000 |  |  |  | 0.000 |  |  |  | 0.000 |  | 0.000 |  | 0.000 |  | 0.000 |  | 0.002 |  | 0.000 |
| **SHAFFER post-hoc** | Original vs. *ADASYN* | **-** | 0.010 |  |  |  | 0.060 |  |  |  | 0.198 |  | 0.168 | - | 0.050 | - | 0.001 |  | 0.060 | - | 0.010 |
|  | Original vs. *ADOMS* |  | 0.391 |  |  | - | 0.001 |  |  | - | 0.003 | - | 0.013 |  | 1.000 |  | 0.946 |  | 0.072 |  | 0.391 |
|  | Original vs. *ROS* | **-** | 0.000 |  |  | - | 0.010 |  |  | - | 0.002 | - | 0.002 | - | 0.000 |  | 1.000 | - | 0.003 | - | 0.000 |
|  | Original vs. *SPIDER* | **-** | 0.000 |  |  | - | 0.005 |  |  | **-** | 0.003 | - | 0.000 | - | 0.000 |  | 0.746 | - | 0.050 | - | 0.000 |
|  | Original vs. *bSMOTE* | **-** | 0.000 |  |  | - | 0.001 |  |  | **-** | 0.000 | - | 0.001 |  | 0.162 |  | 0.060 | - | 0.002 | - | 0.001 |
|  | *ADASYN* vs. *ADOMS* |  | 1.000 |  |  |  | 1.000 |  |  |  | 1.000 |  | 1.000 |  | 0.138 |  | 0.102 |  | 1.000 |  | 1.000 |
|  | *ADASYN* vs. *ROS* |  | 1.000 |  |  |  | 1.000 |  |  |  | 1.000 |  | 1.000 |  | 0.332 | + | 0.001 |  | 1.000 |  | 1.000 |
|  | *ADASYN* vs. *SPIDER* |  | 1.000 |  |  |  | 1.000 |  |  |  | 1.000 |  | 0.198 |  | 0.189 |  | 0.143 |  | 1.000 |  | 1.000 |
|  | *ADASYN* vs. *bSMOTE* |  | 1.000 |  |  |  | 1.000 |  |  |  | 0.168 |  | 0.946 |  | 1.000 |  | 1.000 |  | 1.000 |  | 1.000 |
|  | *ADOMS* vs. *ROS* |  | 0.121 |  |  |  | 1.000 |  |  |  | 1.000 |  | 1.000 | - | 0.000 |  | 1.000 |  | 1.000 |  | 0.211 |
|  | *ADOMS* vs. *SPIDER* |  | 0.168 |  |  |  | 1.000 |  |  |  | 1.000 |  | 0.946 | - | 0.000 |  | 1.000 |  | 1.000 |  | 0.168 |
|  | *ADOMS* vs. *bSMOTE* |  | 0.255 |  |  |  | 1.000 |  |  |  | 1.000 |  | 1.000 |  | 0.292 |  | 1.000 |  | 1.000 |  | 0.255 |
|  | *ROS* vs. *SPIDER* |  | 1.000 |  |  |  | 1.000 |  |  |  | 1.000 |  | 1.000 |  | 1.000 |  | 0.946 |  | 1.000 |  | 1.000 |
|  | *ROS* vs. *bSMOTE* |  | 1.000 |  |  |  | 1.000 |  |  |  | 1.000 |  | 1.000 |  | 0.162 |  | 0.086 |  | 1.000 |  | 1.000 |
|  | *SPIDER* vs. *bSMOTE* |  | 1.000 |  |  |  | 1.000 |  |  |  | 1.000 |  | 1.000 |  | 0.070 |  | 1.000 |  | 1.000 |  | 1.000 |

**Supplementary Table 4**

Friedman and post-hoc Shaffer test for the *oversampling* techniques with *Gmean* measure. Shaffer post-hoc comparisons have been indicated only when Friedman test resulted significant (p-values in the first line). Red colour indicates p-values with significant differences according to shaffer post-hoc (p<0.05); “-” (respectively “+”) indicates that the first algorithm has lower (higher) value than the second one.

|  |  | **DT** | | **DA** | | **LR** | | **NB** | | **SVM** | | **KNN** | | **EnsBO** | | **EnsBA** | | **EnsDA** | | **EnsKNN** | |
| --- | --- | --- | --- | --- | --- | --- | --- | --- | --- | --- | --- | --- | --- | --- | --- | --- | --- | --- | --- | --- | --- |
| **FRIEDMAN TEST** | |  | 0.000 |  | 0.001 |  | 0.000 |  |  |  | 0.000 |  | 0.000 |  | 0.000 |  | 0.000 |  | 0.000 |  | 0.000 |
| **SHAFFER post-hoc** | Original vs. *ADASYN* | **-** | 0.000 | - | 0.003 | **-** | 0.000 |  |  | - | 0.000 | - | 0.008 | - | 0.001 | - | 0.002 | - | 0.000 | - | 0.000 |
|  | Original vs. *ADOMS* | - | 0.010 |  | 0.422 | - | 0.000 |  |  | - | 0.000 | - | 0.000 |  | 1.000 |  | 1.000 | - | 0.000 | - | 0.000 |
|  | Original vs. *ROS* | - | 0.001 | - | 0.003 | - | 0.000 |  |  | - | 0.000 | - | 0.002 | - | 0.000 |  | 1.000 | - | 0.000 |  | 0.846 |
|  | Original vs. *SPIDER* |  | 0.060 |  | 0.582 |  | 0.746 |  |  |  | 0.660 |  | 0.660 | - | 0.007 |  | 0.754 |  | 0.811 |  | 0.846 |
|  | Original vs. *bSMOTE* | - | 0.008 | - | 0.007 | - | 0.028 |  |  | - | 0.007 | - | 0.000 | - | 0.028 | - | 0.231 | - | 0.041 | - | 0.008 |
|  | *ADASYN* vs. *ADOMS* |  | 1.000 |  | 0.582 |  | 1.000 |  |  |  | 1.000 |  | 1.000 | + | 0.050 | + | 0.000 |  | 1.000 |  | 0.846 |
|  | *ADASYN* vs. *ROS* |  | 1.000 |  | 1.000 |  | 1.000 |  |  |  | 1.000 |  | 1.000 |  | 0.838 | + | 0.001 |  | 1.000 | + | 0.000 |
|  | *ADASYN* vs. *SPIDER* |  | 0.730 |  | 0.422 | + | 0.008 |  |  |  | 0.100 |  | 0.168 |  | 1.000 |  | 0.100 | + | 0.004 | + | 0.005 |
|  | *ADASYN* vs. *bSMOTE* |  | 1.000 |  | 1.000 |  | 0.582 |  |  |  | 1.000 |  | 1.000 |  | 1.000 |  | 1.000 |  | 0.295 |  | 0.220 |
|  | *ADOMS* vs. *ROS* |  | 1.000 |  | 0.582 |  | 1.000 |  |  |  | 1.000 |  | 1.000 | - | 0.000 |  | 1.000 |  | 1.000 | + | 0.001 |
|  | *ADOMS* vs. *SPIDER* |  | 1.000 |  | 1.000 | + | 0.013 |  |  |  | 0.060 | + | 0.050 |  | 0.138 |  | 0.135 | + | 0.041 |  | 0.118 |
|  | *ADOMS* vs. *bSMOTE* |  | 1.000 |  | 0.677 |  | 0.746 |  |  |  | 1.000 |  | 1.000 |  | 0.391 | - | 0.000 |  | 0.811 |  | 0.846 |
|  | *ROS* vs. *SPIDER* |  | 1.000 |  | 0.422 | + | 0.029 |  |  |  | 1.000 |  | 0.255 |  | 0.498 |  | 0.721 | + | 0.005 |  | 0.481 |
|  | *ROS* vs. *bSMOTE* |  | 1.000 |  | 1.000 |  | 0.838 |  |  |  | 1.000 |  | 1.000 |  | 0.220 | - | 0.011 |  | 0.340 | - | 0.029 |
|  | *SPIDER* vs. *bSMOTE* |  | 1.000 |  | 0.582 |  | 0.746 |  |  |  | 0.582 |  | 0.168 |  | 1.000 |  | 0.582 |  | 0.811 |  | 0.754 |

**Supplementary Table 5**

Friedman and post-hoc Shaffer test for the *oversampling* techniques with *BalACC* measure. Shaffer post-hoc comparisons have been indicated only when Friedman test resulted significant (p-values in the first line). Red colour indicates p-values with significant differences according to shaffer post-hoc (p<0.05); “-” (respectively “+”) indicates that the first algorithm has lower (higher) value than the second one.

|  |  | **DT** | | **DA** | | **LR** | | **NB** | | **SVM** | | **KNN** | | **EnsBO** | | **EnsBA** | | **EnsDA** | | **EnsKNN** | |
| --- | --- | --- | --- | --- | --- | --- | --- | --- | --- | --- | --- | --- | --- | --- | --- | --- | --- | --- | --- | --- | --- |
| **FRIEDMAN TEST** | |  | 0.000 |  | 0.000 |  | 0.000 |  | 0.005 |  | 0.000 |  | 0.000 |  | 0.000 |  | 0.000 |  | 0.000 |  | 0.000 |
| **SHAFFER post-hoc** | Original vs. *ADASYN* | **-** | 0.000 | - | 0.001 | - | 0.000 | - | 0.006 | - | 0.000 | - | 0.001 | - | 0.001 | - | 0.019 | - | 0.000 | - | 0.000 |
|  | Original vs. *ADOMS* | - | 0.003 |  | 0.721 | - | 0.000 | - | 0.023 | - | 0.000 | - | 0.000 |  | 1.000 |  | 1.000 | - | 0.000 | - | 0.001 |
|  | Original vs. *ROS* | - | 0.000 | - | 0.001 | - | 0.000 | - | 0.015 | - | 0.000 | - | 0.001 | - | 0.000 |  | 1.000 | - | 0.000 |  | 0.947 |
|  | Original vs. *SPIDER* |  | 0.143 |  | 0.511 |  | 0.677 | - | 0.034 |  | 0.746 |  | 0.746 | - | 0.003 |  | 1.000 |  | 0.754 |  | 0.498 |
|  | Original vs. *bSMOTE* | - | 0.041 | - | 0.008 | - | 0.028 |  | 0.422 | - | 0.013 | - | 0.001 | - | 0.028 |  | 0.340 | - | 0.035 | - | 0.010 |
|  | *ADASYN* vs. *ADOMS* |  | 1.000 |  | 0.121 |  | 1.000 |  | 1.000 |  | 1.000 |  | 1.000 | + | 0.010 | + | 0.000 |  | 1.000 |  | 0.754 |
|  | *ADASYN* vs. *ROS* |  | 1.000 |  | 1.000 |  | 1.000 |  | 1.000 |  | 1.000 |  | 1.000 |  | 0.928 | + | 0.007 |  | 1.000 | + | 0.000 |
|  | *ADASYN* vs. *SPIDER* |  | 0.422 |  | 0.232 | + | 0.008 |  | 1.000 |  | 0.086 |  | 0.198 |  | 1.000 |  | 0.340 | + | 0.007 | + | 0.002 |
|  | *ADASYN* vs. *bSMOTE* |  | 0.746 |  | 1.000 |  | 0.582 |  | 1.000 |  | 1.000 |  | 1.000 |  | 1.000 |  | 1.000 |  | 0.391 |  | 0.340 |
|  | *ADOMS* vs. *ROS* |  | 1.000 |  | 0.168 |  | 1.000 |  | 1.000 |  | 1.000 |  | 1.000 | - | 0.000 |  | 1.000 |  | 1.000 | + | 0.002 |
|  | *ADOMS* vs. *SPIDER* |  | 1.000 |  | 1.000 | + | 0.007 |  | 1.000 |  | 0.072 | + | 0.019 | - | 0.028 |  | 0.168 | + | 0.019 |  | 0.100 |
|  | *ADOMS* vs. *bSMOTE* |  | 1.000 |  | 0.511 |  | 0.511 |  | 1.000 |  | 1.000 |  | 1.000 |  | 0.118 | - | 0.013 |  | 0.640 |  | 0.947 |
|  | *ROS* vs. *SPIDER* |  | 0.422 |  | 0.232 | + | 0.050 |  | 1.000 |  | 0.086 |  | 0.198 |  | 0.811 |  | 1.000 | + | 0.008 |  | 0.754 |
|  | *ROS* vs. *bSMOTE* |  | 0.746 |  | 1.000 |  | 1.000 |  | 1.000 |  | 1.000 |  | 1.000 |  | 0.255 |  | 0.232 |  | 0.448 | - | 0.016 |
|  | *SPIDER* vs. *bSMOTE* |  | 1.000 |  | 0.721 |  | 0.640 |  | 1.000 |  | 0.746 |  | 0.198 |  | 1.000 |  | 1.000 |  | 0.640 |  | 0.498 |

**Supplementary Table 6**

Friedman and post-hoc Shaffer test for the *oversampling* techniques with *AUC* measure. Shaffer post-hoc comparisons have been indicated only when Friedman test resulted significant (p-values in the first line). Red colour indicates p-values with significant differences according to shaffer post-hoc (p<0.05); “-” (respectively “+”) indicates that the first algorithm has lower (higher) value than the second one.

|  |  | **DT** | | **DA** | | **LR** | | **NB** | | **SVM** | | **KNN** | | **EnsBO** | | **EnsBA** | | **EnsDA** | | **EnsKNN** | |
| --- | --- | --- | --- | --- | --- | --- | --- | --- | --- | --- | --- | --- | --- | --- | --- | --- | --- | --- | --- | --- | --- |
| **FRIEDMAN TEST** | |  | **0.000** |  |  |  |  |  |  |  | **0.000** |  | **0.035** |  |  |  | **0.000** |  |  |  | **0.000** |
| **SHAFFER post-hoc** | Original vs. *CNNTL* | - | *0.000* |  |  |  |  |  |  | - | *0.001* |  | 0.168 |  |  |  | 0.811 |  |  |  | 1.000 |
|  | Original vs. *NCL* | - | *0.023* |  |  |  |  |  |  |  | 1.000 |  | 0.831 |  |  |  | 0.746 |  |  |  | 0.189 |
|  | Original vs. *OSS* | - | *0.008* |  |  |  |  |  |  |  | 0.168 |  | 0.831 |  |  |  | 0.811 |  |  |  | 0.640 |
|  | Original vs. *RUS* | - | *0.000* |  |  |  |  |  |  | - | *0.000* | - | *0.023* |  |  |  | 0.189 |  |  | - | *0.042* |
|  | Original vs. *SBC* | - | *0.019* |  |  |  |  |  |  | - | *0.019* |  | 1.000 |  |  |  | 0.340 |  |  |  | 0.162 |
|  | *CNNTL* vs. *NCL* |  | 1.000 |  |  |  |  |  |  | + | *0.041* |  | 1.000 |  |  | - | *0.019* |  |  | - | *0.015* |
|  | *CNNTL* vs. *OSS* |  | 1.000 |  |  |  |  |  |  |  | 0.746 |  | 1.000 |  |  |  | 1.000 |  |  |  | 1.000 |
|  | *CNNTL* vs. *RUS* |  | 1.000 |  |  |  |  |  |  |  | 1.000 |  | 1.000 |  |  | - | *0.003* |  |  | - | *0.002* |
|  | *CNNTL* vs. *SBC* |  | 1.000 |  |  |  |  |  |  |  | 1.000 |  | 1.000 |  |  | - | *0.005* |  |  | - | *0.013* |
|  | *NCL* vs. *OSS* |  | 1.000 |  |  |  |  |  |  |  | 0.754 |  | 1.000 |  |  | + | 0.023 |  |  | + | *0.001* |
|  | *NCL* vs. *RUS* |  | 1.000 |  |  |  |  |  |  | - | *0.002* |  | 1.000 |  |  |  | 1.000 |  |  |  | 1.000 |
|  | *NCL* vs. *SBC* |  | 1.000 |  |  |  |  |  |  |  | 0.255 |  | 1.000 |  |  |  | 1.000 |  |  |  | 1.000 |
|  | *OSS* vs. *RUS* |  | 1.000 |  |  |  |  |  |  |  | 0.168 |  | 1.000 |  |  | - | *0.003* |  |  | - | *0.000* |
|  | *OSS* vs. *SBC* |  | 1.000 |  |  |  |  |  |  |  | 1.000 |  | 1.000 |  |  | - | *0.007* |  |  | - | *0.001* |
|  | *RUS* vs. *SBC* |  | 1.000 |  |  |  |  |  |  |  | 0.746 |  | 0.314 |  |  |  | 1.000 |  |  |  | 1.000 |

**Supplementary Table 7**

Friedman and post-hoc Shaffer test for the *undersampling* techniques with *Fm* measure. Shaffer post-hoc comparisons have been indicated only when Friedman test resulted significant (p-values in the first line). Red colour indicates p-values with significant differences according to shaffer post-hoc (p<0.05); “-” (respectively “+”) indicates that the first algorithm has lower (higher) value than the second one.

|  |  | **DT** | | **DA** | | **LR** | | **NB** | | **SVM** | | **KNN** | | **EnsBO** | | **EnsBA** | | **EnsDA** | | **EnsKNN** | |
| --- | --- | --- | --- | --- | --- | --- | --- | --- | --- | --- | --- | --- | --- | --- | --- | --- | --- | --- | --- | --- | --- |
| **FRIEDMAN TEST** | |  | **0.000** |  | **0.013** |  | **0.000** |  |  |  | **0.000** |  | **0.000** |  | **0.000** |  | **0.000** |  | **0.000** |  | **0.000** |
| **SHAFFER post-hoc** | Original vs. *CNNTL* | - | *0.003* |  | 0.090 | - | *0.000* |  |  | - | *0.000* | - | *0.000* | - | *0.000* |  | 0.232 | - | *0.000* | - | *0.004* |
|  | Original vs. *NCL* | - | *0.002* |  | 0.143 |  | 0.220 |  |  |  | 0.754 |  | 0.481 | - | *0.003* | - | *0.005* | - | *0.041* |  | 0.220 |
|  | Original vs. *OSS* | - | *0.028* |  | 1.000 |  | 0.060 |  |  | - | *0.013* | - | *0.015* |  | 0.102 |  | 0.121 |  | 0.270 | - | *0.023* |
|  | Original vs. *RUS* | - | *0.000* |  | 0.232 | - | *0.000* |  |  | - | *0.000* | - | *0.000* | - | *0.000* | - | *0.000* | - | *0.000* | - | *0.000* |
|  | Original vs. *SBC* |  | 0.232 |  | 1.000 |  | 0.566 |  |  | - | *0.028* | - | *0.034* |  | 0.511 |  | 0.232 |  | 0.270 | - | *0.002* |
|  | *CNNTL* vs. *NCL* |  | 1.000 |  | 1.000 |  | 0.391 |  |  | + | *0.008* | + | *0.003* |  | 1.000 |  | 1.000 |  | 0.606 |  | 0.677 |
|  | *CNNTL* vs. *OSS* |  | 1.000 |  | 1.000 |  | 0.754 |  |  |  | 0.754 |  | 0.340 |  | 0.295 |  | 1.000 |  | 0.270 |  | 1.000 |
|  | *CNNTL* vs. *RUS* |  | 1.000 |  | 1.000 |  | 1.000 |  |  |  | 0.947 |  | 1.000 |  | 1.000 |  | 0.168 |  | 1.000 |  | 0.391 |
|  | *CNNTL* vs. *SBC* |  | 1.000 |  | 0.090 |  | 0.168 |  |  |  | 0.481 |  | 0.189 | + | *0.050* |  | 1.000 |  | 0.270 |  | 1.000 |
|  | *NCL* vs. *OSS* |  | 1.000 |  | 1.000 |  | 1.000 |  |  |  | 0.295 |  | 0.426 |  | 1.000 |  | 1.000 |  | 1.000 |  | 1.000 |
|  | *NCL* vs. *RUS* |  | 1.000 |  | 1.000 |  | 0.086 |  |  | - | *0.000* | - | *0.003* |  | 1.000 |  | 1.000 |  | 0.270 | - | *0.010* |
|  | *NCL* vs. *SBC* |  | 1.000 |  | 0.198 |  | 1.000 |  |  |  | 0.438 |  | 0.508 |  | 0.511 |  | 1.000 |  | 1.000 |  | 0.660 |
|  | *OSS* vs. *RUS* |  | 0.340 |  | 1.000 |  | 0.295 |  |  |  | 0.295 |  | 0.340 |  | 0.511 |  | 0.232 |  | 0.072 |  | 0.168 |
|  | *OSS* vs. *SBC* |  | 1.000 |  | 1.000 |  | 1.000 |  |  |  | 0.947 |  | 1.000 |  | 1.000 |  | 1.000 |  | 1.000 |  | 1.000 |
|  | *RUS* vs. *SBC* |  | 0.072 |  | 0.314 | + | *0.019* |  |  |  | 0.162 |  | 0.189 |  | 0.121 |  | 0.121 |  | 0.072 |  | 0.660 |

**Supplementary Table 8**

Friedman and post-hoc Shaffer test for the *underampling* techniques with *Gmean* measure. Shaffer post-hoc comparisons have been indicated only when Friedman test resulted significant (p-values in the first line). Red colour indicates p-values with significant differences according to shaffer post-hoc (p<0.05); “-” (respectively “+”) indicates that the first algorithm has lower (higher) value than the second one.

|  |  | **DT** | | **DA** | | **LR** | | **NB** | | **SVM** | | **KNN** | | **EnsBO** | | **EnsBA** | | **EnsDA** | | **EnsKNN** | |
| --- | --- | --- | --- | --- | --- | --- | --- | --- | --- | --- | --- | --- | --- | --- | --- | --- | --- | --- | --- | --- | --- |
| **FRIEDMAN TEST** | |  | **0.000** |  | **0.000** |  | **0.000** |  | **0.009** |  | **0.000** |  | **0.000** |  | **0.000** |  | **0.000** |  | **0.000** |  | **0.000** |
| **SHAFFER post-hoc** | Original vs. *CNNTL* | - | *0.000* | - | *0.034* | - | *0.000* |  | 0.060 | - | *0.000* | - | *0.000* | - | *0.000* | - | *0.003* | - | *0.000* | - | *0.000* |
|  | Original vs. *NCL* |  | 0.162 |  | 0.255 |  | 0.769 |  | 0.558 |  | 0.754 |  | 0.754 |  | 0.295 |  | 0.143 |  | 0.295 |  | 0.511 |
|  | Original vs. *OSS* |  | 0.377 |  | 1.000 |  | 0.085 |  | 1.000 |  | 0.162 |  | 0.162 |  | 0.391 |  | 0.143 |  | 0.481 |  | 0.102 |
|  | Original vs. *RUS* | - | *0.000* | - | *0.001* | - | *0.000* |  | 0.168 | - | *0.000* | - | *0.000* | - | *0.000* | - | *0.000* | - | *0.000* | - | *0.000* |
|  | Original vs. *SBC* | - | *0.003* |  | 0.102 | - | *0.003* |  | 0.582 | - | *0.003* | - | *0.003* | - | *0.003* | - | *0.001* | - | *0.001* | - | *0.001* |
|  | *CNNTL* vs. *NCL* |  | 0.220 |  | 1.000 | + | 0.002 |  | 1.000 | + | *0.000* | + | *0.001* | + | *0.024* |  | 1.000 | + | 0.029 |  | 0.102 |
|  | *CNNTL* vs. *OSS* |  | 0.060 |  | 0.060 |  | 0.118 | + | *0.051* | + | *0.035* | + | *0.020* | + | *0.023* |  | 1.000 | + | 0.008 |  | 0.511 |
|  | *CNNTL* vs. *RUS* |  | 0.769 |  | 1.000 |  | 0.769 |  | 1.000 |  | 0.811 |  | 0.811 |  | 1.000 |  | 0.391 |  | 1.000 |  | 0.696 |
|  | *CNNTL* vs. *SBC* |  | 0.874 |  | 1.000 |  | 0.754 |  | 1.000 |  | 0.541 |  | 0.377 |  | 0.508 |  | 1.000 |  | 1.000 |  | 0.874 |
|  | *NCL* vs. *OSS* |  | 0.874 |  | 0.391 |  | 0.677 |  | 0.365 |  | 0.754 |  | 0.754 |  | 1.000 |  | 1.000 |  | 1.000 |  | 0.874 |
|  | *NCL* vs. *RUS* | - | *0.010* |  | 0.340 | - | *0.000* |  | 1.000 | - | *0.000* | - | *0.000* | - | *0.007* | - | *0.019* | - | *0.005* | - | *0.002* |
|  | *NCL* vs. *SBC* |  | 0.677 |  | 1.000 |  | 0.100 |  | 1.000 |  | 0.060 |  | 0.162 |  | 0.481 |  | 1.000 |  | 0.335 |  | 0.220 |
|  | *OSS* vs. *RUS* | - | *0.001* | - | *0.001* | - | *0.034* |  | 0.102 | - | *0.023* | - | *0.013* | - | *0.004* | - | *0.019* | - | *0.001* | - | *0.028* |
|  | *OSS* vs. *SBC* |  | 0.340 |  | 0.168 |  | 0.769 |  | 0.558 |  | 0.754 |  | 0.754 |  | 0.391 |  | 1.000 |  | 0.118 |  | 0.677 |
|  | *RUS* vs. *SBC* |  | 0.340 |  | 0.811 |  | 0.384 |  | 1.000 |  | 0.498 |  | 0.335 |  | 0.391 |  | 0.582 |  | 0.481 |  | 0.511 |

**Supplementary Table 9**

Friedman and post-hoc Shaffer test for the *undersampling* techniques with *BalACC* measure. Shaffer post-hoc comparisons have been indicated only when Friedman test resulted significant (p-values in the first line). Red colour indicates p-values with significant differences according to shaffer post-hoc (p<0.05); “-” (respectively “+”) indicates that the first algorithm has lower (higher) value than the second one.

|  |  | **DT** | | **DA** | | **LR** | | **NB** | | **SVM** | | **KNN** | | **EnsBO** | | **EnsBA** | | **EnsDA** | | **EnsKNN** | |
| --- | --- | --- | --- | --- | --- | --- | --- | --- | --- | --- | --- | --- | --- | --- | --- | --- | --- | --- | --- | --- | --- |
| **FRIEDMAN TEST** | |  | **0.000** |  | **0.000** |  | **0.000** |  | **0.004** |  | **0.000** |  | **0.000** |  | **0.000** |  | **0.000** |  | **0.000** |  | **0.000** |
| **SHAFFER post-hoc** | Original vs. *CNNTL* | - | 0.001 | - | 0.023 | - | 0.000 | - | 0.042 | - | 0.000 | - | 0.000 | - | 0.000 |  | 0.060 | - | 0.000 | - | 0.003 |
|  | Original vs. *NCL* |  | 0.102 |  | 0.448 |  | 1.017 |  | 0.660 |  | 0.846 |  | 0.498 |  | 0.138 |  | 0.162 |  | 0.295 |  | 0.295 |
|  | Original vs. *OSS* |  | 0.842 |  | 1.420 |  | 0.189 |  | 2.363 |  | 0.511 |  | 0.255 |  | 0.448 |  | 0.220 |  | 0.677 |  | 0.220 |
|  | Original vs. *RUS* | - | 0.000 | - | 0.001 | - | 0.000 |  | 0.060 | - | 0.000 | - | 0.000 | - | 0.000 | - | 0.000 | - | 0.000 | - | 0.000 |
|  | Original vs. *SBC* | - | 0.000 |  | 0.102 | - | 0.003 |  | 0.143 | - | 0.013 | - | 0.003 | - | 0.005 | - | 0.000 | - | 0.001 | - | 0.000 |
|  | *CNNTL* vs. *NCL* |  | 0.842 |  | 0.928 | + | 0.004 |  | 1.320 | + | 0.003 | + | 0.008 |  | 0.060 |  | 1.533 |  | 0.071 |  | 0.438 |
|  | *CNNTL* vs. *OSS* |  | 0.138 | + | 0.019 |  | 0.162 | + | 0.050 | + | 0.020 | + | 0.035 | + | 0.019 |  | 1.533 | + | 0.015 |  | 0.541 |
|  | *CNNTL* vs. *RUS* |  | 0.842 |  | 1.239 |  | 1.017 |  | 2.363 |  | 1.022 |  | 1.181 |  | 1.265 |  | 0.102 |  | 1.312 |  | 0.438 |
|  | *CNNTL* vs. *SBC* |  | 0.842 |  | 1.420 |  | 1.017 |  | 2.363 |  | 0.541 |  | 0.628 |  | 0.541 |  | 0.377 |  | 1.312 |  | 0.929 |
|  | *NCL* vs. *OSS* |  | 0.842 |  | 0.391 |  | 0.838 |  | 0.946 |  | 1.022 |  | 1.181 |  | 1.265 |  | 1.533 |  | 1.312 |  | 0.929 |
|  | *NCL* vs. *RUS* | - | 0.041 |  | 0.189 | - | 0.000 |  | 1.692 | - | 0.001 | - | 0.001 | - | 0.028 | - | 0.023 | - | 0.008 | - | 0.007 |
|  | *NCL* vs. *SBC* |  | 0.842 |  | 1.420 | - | 0.050 |  | 1.749 |  | 0.220 |  | 0.255 |  | 0.769 |  | 0.220 |  | 0.295 |  | 0.086 |
|  | *OSS* vs. *RUS* | - | 0.001 | - | 0.001 | - | 0.028 |  | 0.102 | - | 0.010 | - | 0.010 | - | 0.005 | - | 0.013 | - | 0.001 | - | 0.010 |
|  | *OSS* vs. *SBC* |  | 0.102 |  | 0.086 |  | 0.721 |  | 0.232 |  | 0.541 |  | 0.498 |  | 0.448 |  | 0.138 |  | 0.060 |  | 0.086 |
|  | *RUS* vs. *SBC* |  | 0.842 |  | 0.811 |  | 0.721 |  | 2.363 |  | 0.511 |  | 0.498 |  | 0.448 |  | 1.480 |  | 0.754 |  | 0.929 |
